# Highly efficient baculovirus-mediated multigene delivery in primary cells

**DOI:** 10.1038/ncomms11529

**Published:** 2016-05-04

**Authors:** Maysam Mansouri, Itxaso Bellon-Echeverria, Aurélien Rizk, Zahra Ehsaei, Chiara Cianciolo Cosentino, Catarina S. Silva, Ye Xie, Frederick M. Boyce, M. Wayne Davis, Stephan C. F. Neuhauss, Verdon Taylor, Kurt Ballmer-Hofer, Imre Berger, Philipp Berger

**Affiliations:** 1 Biomolecular Research, Molecular Cell Biology, Paul Scherrer Institute, CH-5232 Villigen, Switzerland; 2 European Molecular Biology Laboratory (EMBL), Grenoble Outstation, B.P. 181, 38042 Grenoble Cedex 9, France; 3 Department of Biomedicine, University of Basel, CH-4058 Basel, Switzerland; 4 Institute of Molecular Life Sciences, University of Zürich, CH-8057 Zürich, Switzerland; 5 Department of Neurology, Massachusetts General Hospital, Cambridge, Massachusetts 02139, USA; 6 Department of Biology and Howard Hughes Medical Institute, University of Utah, Salt Lake City, Utah 84112-0840, USA; 7 School of Biochemistry, University of Bristol, Bristol BS8 1TD, UK

## Abstract

Multigene delivery and subsequent cellular expression is emerging as a key technology required in diverse research fields including, synthetic and structural biology, cellular reprogramming and functional pharmaceutical screening. Current viral delivery systems such as retro- and adenoviruses suffer from limited DNA cargo capacity, thus impeding unrestricted multigene expression. We developed MultiPrime, a modular, non-cytotoxic, non-integrating, baculovirus-based vector system expediting highly efficient transient multigene expression from a variety of promoters. MultiPrime viruses efficiently transduce a wide range of cell types, including non-dividing primary neurons and induced-pluripotent stem cells (iPS). We show that MultiPrime can be used for reprogramming, and for genome editing and engineering by CRISPR/Cas9. Moreover, we implemented dual-host-specific cassettes enabling multiprotein expression in insect and mammalian cells using a single reagent. Our experiments establish MultiPrime as a powerful and highly efficient tool, to deliver multiple genes for a wide range of applications in primary and established mammalian cells.

## Abstract (web summary)

Current viral gene delivery systems are limited in the amount of foreign DNA they can deliver to cells. Here the authors develop MultiPrime, a baculovirus-based vector system capable of multigene delivery into a wide variety of cells, and use Multiprime for genome engineering by CRISPR/Cas9.

## Graphical abstract

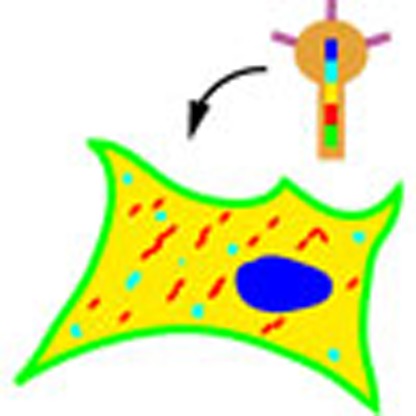

Multigene delivery into cultured cells or tissues is emerging as an indispensable tool for many applications in biological research and development. Examples include simultaneous labelling of living cells with various fluorescently-tagged sensors for monitoring changes in cellular architecture or metabolism, lineage tracing during morphogenesis to follow regenerative tissue processes, visualization of multicomponent molecular pathways for high-content screening in pharmacological applications or the construction of recombinant adeno-associated viruses for gene therapy^1^,^2^,^3^,^4^,^5^. Multigene delivery systems also allow reprogramming of somatic cells to stem cells^6^ or to specifically differentiated cell lines^7^. The construction of complex multigene circuits in mammalian cells is a core concept in synthetic biology requiring the flexible generation of modular multigene expression systems^8^,^9^. Moreover, structural and biophysical characterization of multiprotein complexes relies on co-expression of an ensemble of genes that may include ancillary factors, such as chaperones or protein modifying enzymes^10^. All applications share in common that they require versatile tool-kits to flexibly engineer and to simultaneously, efficiently and reproducibly deliver multiple genes into target host cells.

Several strategies for multigene expression in mammalian cells exist, each with its own merits^11^. All of these applications require specific boundary conditions. For instance, it is essential that all transfected cells in a population express all heterologous genes at the same defined level, on an equal time frame. Other applications require that the proteins of interest retain native N- or C termini. Furthermore, long-term stable expression versus transient expression is a crucial parameter to be considered. Ideally, an efficient multigene-delivery system would provide the means to afford many or all of these requirements.

We have developed systems for the delivery of multigene constructs in prokaryotic and eukaryotic hosts^12^,^13^,^14^. A central feature of these technologies is the assembly of multiple gene expression cassettes by recombineering^15^, from custom designed plasmids encoding specific genes, into a single multicomponent DNA construct for gene delivery. This approach was shown to overcome the limitations hampering classical co-transfection or co-infection techniques, which for statistical reasons, are inherently unbalanced^16^,^17^. More recently, we introduced MultiLabel^14^ and demonstrated that homogenous mammalian cell populations could be achieved by transient introduction of single recombineering-based multigene expression plasmids by classical transfection methods. This method performs well with cell lines that are readily transfected, such as HEK293 or HeLa cells. However, a large number of cell lines and particularly primary cells are markedly recalcitrant to plasmid transfection, thus requiring a different approach. Primary cells are a central focus of contemporary biological research efforts, and efficient multigene delivery in primary cells is thus highly desirable.

Infection by viral vectors emerged as the dominant method of choice to deliver genes into primary cells^18^. An ideal viral vector for multigene delivery should have virtually unlimited foreign DNA cargo capacity allowing for integration of a multitude of independent expression cassettes, functionalities and regulatory elements. Moreover, such an optimal viral vector should exhibit low cytotoxicity in mammalian cells and should enable transduction of dividing and non-dividing mammalian cells alike. Currently used lenti- and other retroviruses, as well as adeno- and adeno-associated viruses have a limitation on DNA cargo size due to spatial constraints imposed by the tight geometry of their capsids.

Baculoviral vectors, in contrast, can accommodate very large DNA cargo insertions^19^. The *Autographa californica* multiple nuclear polyhedrovirus (AcMNPV), is a baculovirus with a large (134 kb) double-stranded circular DNA genome that normally infects specific moth larvae^19^. Transgene capacity of AcMNPV is very large, extending probably beyond 100 kbp. Replication of AcMNPV is highly insect-cell specific; however, AcMNPV is capable of efficiently transducing not only insect but also mammalian cells. Transduction is usually transient without DNA integration into the target cell genome and such viruses are replication deficient^20^,^21^. In baculoviruses used for mammalian cell transduction (BacMam)^22^,^23^ heterologous genes are placed under the control of mammalian promoters and inserted into the baculoviral genome, and viral stocks are produced in insect cells. Once the baculovirus enters mammalian cells, these genes are actively transcribed within 9 h and the cells produce the heterologous gene product. In the last decade, baculovirus has emerged as a useful and safe technology to deliver heterologous genetic material to mammalian cell types both *in vitro* and *in vivo*^24^.

Here we introduce MultiPrime, a novel tool-kit specifically designed for efficient delivery of large multigene constructs into primary and established mammalian cells. MultiPrime enables simultaneous expression of multiple, independent cassettes in mammalian cells. This system combines the ease and flexibility of our recombination-mediated multigene DNA assembly technology with the superior performance of baculovirus as a viral vector for transducing mammalian cells. We transduced with MultiPrime a large variety of cell types including difficult to transfect stem cells and primary neurons. Moreover, we utilized MultiPrime for reprogramming mouse embryo fibroblasts (MEFs) into neurons. Further, we show that our system is not limited to mammalian transduction but can also be used to transduce zebrafish embryos. We applied our system to create synthetic multicomponent intracellular biosensor systems, such as Rab GTPases regulating vesicular membrane traffic in cells, phosphoinositide binding signalling proteins or fluorescently-labelled cytoskeletal markers. These biosensors were simultaneously delivered and expressed in mammalian cells allowing to quantitatively monitor a large variety of intracellular parameters. A wide range of promoters are available in MultiPrime, to regulate and fine-tune individual heterologous target gene expression.

With the objective to provide a means to concomitantly exploit with one single expression system the advantages of high-level protein production in baculovirus-infected insect cells and protein expression in a ‘native' mammalian environment, we incorporated dual-host-specific promoters in MultiPrime that are functional in both insect and mammalian cells.

Our MultiPrime system is not limited to the assembly of RNA polymerase II-based expression cassettes. It can likewise harbour U6-driven expression cassettes and homology constructs that are required for CRISPR/Cas9-mediated genome engineering. We demonstrate the aptitude of our system by applying MultiPrime-CRISPR/Cas9 to express a HMGA1-EGFP fusion protein in primary cells from the native genomic HMGA locus.

## Results

### MultiPrime system design

We developed MultiPrime specifically to overcome the limitations of transfecting mammalian cells for multigene transfer applications. We redesigned our previous pFL plasmid^25^, which contains the Tn7R and Tn7L DNA elements required for Tn7-transposase-mediated integration into a baculovirus genome containing a Tn7-attachment site^26^. Expression cassettes with promoters active in mammalian cells, or, alternatively, with activity in both mammalian and insect cells, were inserted into this MultiPrime acceptor plasmid. This acceptor plasmid is poised to receive further multigene expression cassettes by incorporating donor plasmids (Supplementary Figs 1–3). All donors from our previous plasmid-based MultiLabel system can be used for incorporation into this new acceptor to generate acceptor-donor fusions by recombineering. Moreover, expression cassettes can be freely exchanged between donors and acceptors due to the modular design^14^.

Acceptors or acceptor–donor fusions containing multigene expression cassettes are integrated into baculoviral genomes by means of Tn7 transposition^25^. We utilized two different baculoviral genomes in our experiments. In addition to our EMBacY baculoviral genome^25^ we generated in this study a new baculoviral genome, MultiBacMam, which expresses a vesicular stomatitis virus glycoprotein (VSV-G) and the fluorescent protein mCherry in insect cells during virus production. Both genomes were generated from the original MultiBac virus by integrating genes encoding EYFP (EMBacY) or mCherry and VSV-G (MultiBacMam) by Cre recombination into the LoxP site present on the MultiBac virus^12^,^25^. The expression of mCherry results in a characteristic purple colour of MultiBacMam infected cell cultures (Supplementary Fig. 1), thereby simplifying the tracking of virus amplification by eye. Moreover, the MultiBacMam virus gives rise to baculovirions displaying VSV-G on their surface. The presence of VSV-G in the baculoviral envelope has been shown to increase the efficacy of mammalian cell transduction^27^. All composite baculoviruses prepared in this study were produced in Sf21 cells. Virus was obtained with comparable efficiency and resulting in similar viral titers to what we had observed in previous multigene expressions in insect cells with recombinant MultiBac or EMBacY viruses^25^. Our multigene viruses are characterized by multiple use of regulatory elements such as the CMV promoter, which in theory could introduce genomic instability during repeated rounds of viral amplicfication^14^,^25^. We had developed previously efficient protocols to amplify baculoviral constructs containing multiple copies of late viral promoters (polh and p10) by stringently applying a low multiplicity of infection (MOI) regimen and few, ideally not more than two amplification rounds thus preventing accumulation of non-productive viruses containing genomic deletions^28^. Strictly adhering to this strategy for amplifying baculoviruses containing multiple copies of CMV promoter-driven expression cassettes again resulted in stable virus producing all proteins of choice in each transduced mammalian cell in homogenous cultures, while failure to adhere to the protocol resulted in heterogeneous cell populations where expression of individual heterologous genes had been lost, presumably due to accumulation of deletion virus species (Supplementary Fig. 4). Occasionally, we observed cell fusion in tissue culture plates of insect cells infected with MultiBacMam viruses, which, however, was found not to be detrimental to transduction experiments with the viral supernatant. In the following, we use the EMBacY baculoviral genome to prepare composite MultiPrime baculoviruses, unless indicated otherwise.

### Transduction and viability

As proof-of-concept, we generated a composite MultiPrime baculovirus expressing five fluorescently-tagged proteins (EBFP2-nucleus, mTFP1-FYVE (PI-3-P-binding, early endosomes), EYFP-tubulin, Mito-dsRED (mitochondria) and Plum-PLCδ-PH (PI-4,5-P_2_-binding, plasma membrane) localizing to different subcellular compartments. We used this baculovirus to test a variety of different cell types for their propensity to be transduced efficiently. We initially transduced well-established cell lines including HEK293, PAE, COS7, HeLa, SK-MEL-28, CCL39 and Swiss 3T3. All cell lines that were tested proved to be highly transduceable by MultiPrime and efficiently expressed all heterologous proteins (Supplementary Fig. 5 and data not shown). Typically, transduction efficiencies ranged between 20 and 100%. Transduction frequencies up to 100% were observed even in cell lines, such as PAE that are traditionally considered to be difficult to transfect.

**Figure 1:**
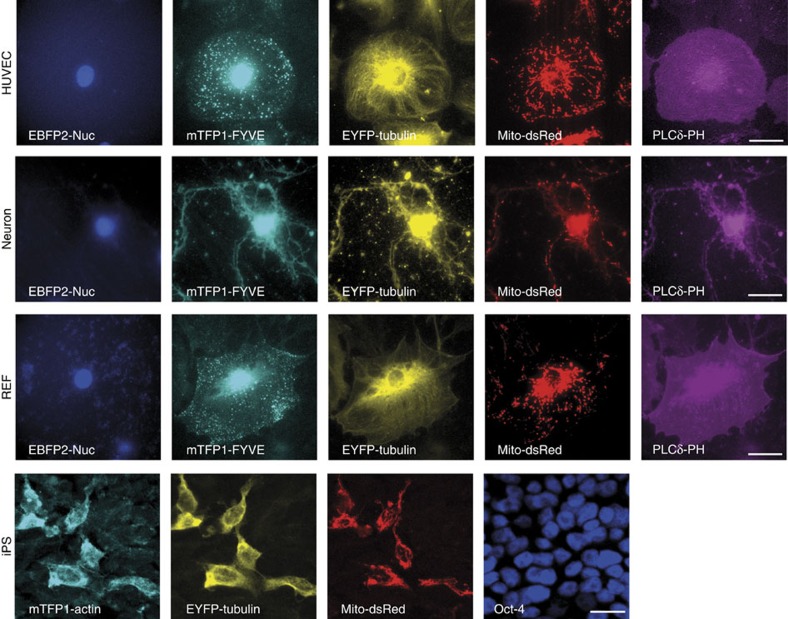
Multigene expression in primary cells by MultiPrime. HUVEC, REF cells and rat cortical neurons were infected with a MultiPrime baculovirus encoding EBFP2-Nuc (labelling the nucleus), mTFP1-FYVE (PI-3-P containing endosomes), tubulin-EYFP (cytoskeleton), Mito-dsRed (mitochondria) and PLCδ-PH (PI-4,5-P2; plasma membrane). iPS cells were transduced with a virus encoding mTFP1-actin, EYFP-tubulin and Mito-dsRed. Oct4 was used as a marker for pluripotent stem cells. All infected cells express all heterologous proteins. Scale bar, 20 μm.

We then asked whether we can use MultiPrime to transfect primary cells. Primary cells are an intense focus of contemporary research efforts for many reasons, and it is well documented that they are typically extremely difficult to transfect. For this experiment, we utilized human umbilical vein endothelial cells (HUVEC), rat embryo fibroblasts (REF), rat cortical neurons and human-induced pluripotent stem cells (iPS). With our MultiPrime virus, all of these primary cell types could be transduced efficiently and again expressed all heterologous genes of interest, compellingly underscoring the utility of our approach (Fig. 1 and Supplementary Fig. 5).

**Figure 2:**
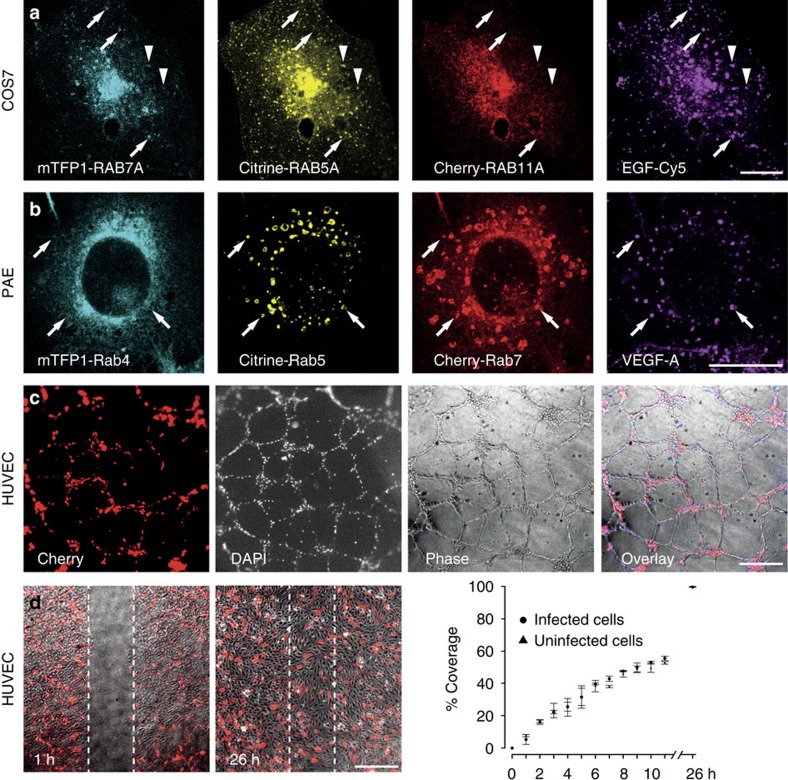
Infected cells retain functionality. (**a**) COS7 cells were infected with MultiPrime baculoviruses expressing the indicated fluorescently**-**tagged RAB GTPases. Cells were stimulated for 3 h with Cy5-labelled EGF. As expected, EGF was found in RAB7A vesicles (arrows) and RAB5A vesicles (arrowheads) but not in RAB11 vesicles. A quantitative time-resolved analysis is provided (Supplementary Movie 1). (**b**) PAE cells stably expressing VEGFR2 and neuropilin-1 were infected with baculoviruses expressing the indicated fluorescently-tagged RAB GTPases. Cells were then stimulated with Nt647-labelled VEGF. VEGF can be found in RAB5 and Rab7 vesicles (arrows). (**c**) Tube formation: HUVEC were infected with a baculovirus expressing mTFP1-actin, EYFP-tubulin and Mito-dsRed (only red channel is shown). DAPI was used to counterstain nuclei of all cells. Approximately 30% of cells were infected. Infected and uninfected cells contribute to tubes. (**d**) Migration: HUVEC were infected with a baculovirus expressing mTFP1-actin, EYFP-tubulin and Mito-dsRed (only red channel is shown). Approximately 30% of cells were infected. Infected and uninfected cells migrate at same rates. Scale bar, 20 μm (**a**,**b**); 500 μm (**c**,**d**).

To analyze viability and functionality, we proceeded to express multiple intracellular sensors simultaneously from a MultiPrime baculovirus. COS7 cells expressing epidermal growth factor receptor (EGFR) endogenously were used to monitor trafficking of EGFR. Genes encoding fluorescently-tagged markers for early (RAB5A), recycling (RAB11A) and late (RAB7A) endosomes were expressed from a single MultiPrime virus and the cells were stimulated 40 h post-transduction with Cy5-labelled epidermal growth factor (EGF). As expected, EGF was found in early (RAB5A+) and late (RAB7A+) endosomes, but not in recycling (RAB11A+) endosomes after 30 min (Fig. 2a). A time-resolved quantitative analysis confirmed that EGF was transferred to late endosomes but not to RAB11-positive, recycling vesicles (Supplementary Movie 1). Next, we used PAE cells stably expressing VEGFR2 and neuropilin-1 (NRP1). A composite MultiPrime baculovirus was used to express fluorescently-tagged Rab4, Rab5 and Rab7 in these cells. Cells were then stimulated with Nt647-labelled VEGF-A165a for 3 h. VEGF was found in Rab5 and Rab7 vesicles clearly indicating that it was properly internalized, in accordance with previous reports^3^. Using the same cell line transduced with the RAB5A-RAB11A-RAB7A virus in a time-resolved study, we could show that VEGFR2 behaves differently from EGFR. VEGFR2 is, in contrast to EGFR, partly recycled through the RAB11 compartment (Supplementary Movie 2).

Next, we tested the functionality of baculovirus-transduced HUVEC in migration and angiogenesis assays. HUVEC transduced with a MultiPrime baculovirus encoding mTFP1-actin, EYFP-tubulin and Mito-DsRed were plated on matrigel and incubated for 14 h. HUVEC are known to establish a characteristic vascular network within this time frame. Transduced cells efficiently integrated in this network, clearly demonstrating that MultiPrime-transduced HUVEC show identical behaviour compared to untransduced cells (Fig. 2c). Moreover, in a migration assay, we could show that transduced cells migrate with similar efficiency as untransduced cells (Fig. 2d).

**Figure 3:**
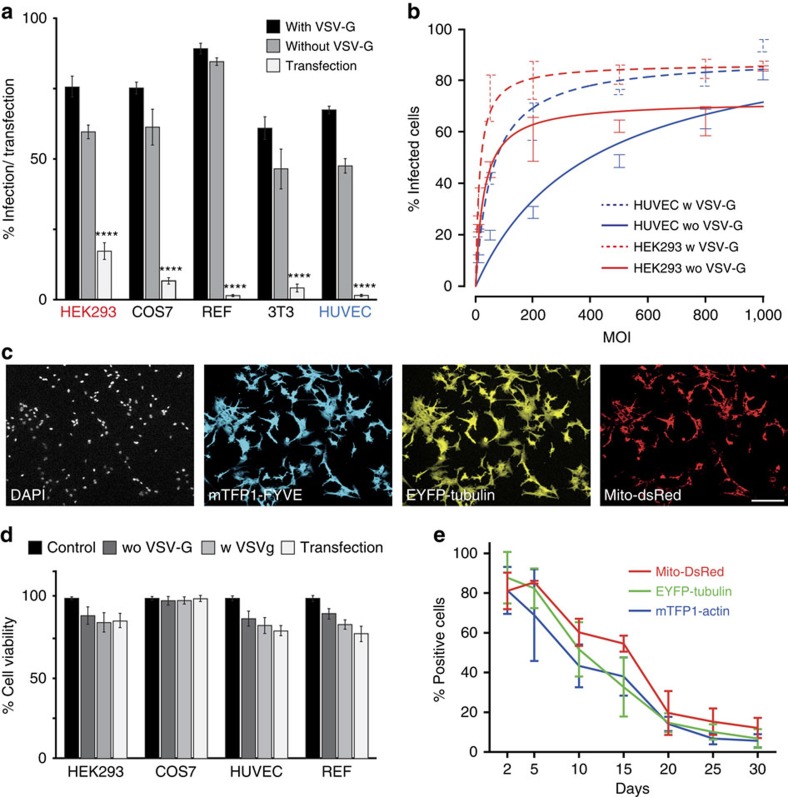
Transduction efficacy. (**a**) Transduction by MultiPrime baculovirus was compared to plasmid-based transfection. MultiBacMam bacoluvirus expressing VSV-G and EMBacY baculovirus devoid of VSV-G were used. For plasmid-based transfection, the plasmid (13 kb) used originally inserted into the recombinant baculoviruses was utilized. MultiPrime-mediated transduction is markedly superior in all cell types tested. Data shows mean value±s.d.; *n*=3; ****P<0.0001 determined by comparing transfection with transduction with or without VSV-G using one way analysis of variance followed by the Dunnet's *post hoc* test. (**b**) Effects of the MOI are shown. (**c**) PAE cells were infected with a MultiPrime baculovirus expressing three proteins at a MOI of 500. DAPI was used to counterstain nuclei of all cells. Virtually all cells are infected and express all heterologous proteins. Scale bar, 100 μm. (**d**) Toxicity of transduction was measured by means of a MTT assay. MultiPrime transduction exhibits comparable, low toxicity as plasmid transfection. (**e**) The persistence of heterologous expression was quantified by fluorescence in REF cells. The percentage of positive cells for each individual protein is shown over time.

We quantitatively compared baculovirus-mediated transduction with the classical plasmid transfection approach. In addition to EMBacY, we used our MultiBacMam baculovirus displaying VSV-G on its surface in this experiment. As expected, we observed increased transduction rates with MultiBacMam compared to EMBacY, albeit the gain in efficacy in our hands turned out to be modest in many cases. Clearly, transduction with both EMBacY and MultiBacMam outperformed the classical transfection approach (Fig. 3a). We routinely obtained transduction efficiencies higher than 50% when using the MultiPrime baculovirus compared to transfection efficiencies well below 20% with the corresponding plasmid (13,305 bp) that had been used to generate the composite baculovirus.

Baculovirus displaying VSV-G on its surface was superior to virus lacking VSV-G at all tested MOI. Saturation was usually obtained at a MOI of 500 (Fig. 3b). The relative expression levels between cells appear to be similar (Fig. 3c). This is in contrast to transfected cells that typically show a wide variety of expression levels (data not shown). Since we use relatively high MOI, the toxicity of the virus could conceivably be an issue. We tested baculovirus toxicity at MOI 500 compared to plasmid transfection with Fugene HD, which is considered to be a mild transfection reagent. Both EMBacY- and MultiBacMam-derived viruses exhibit negligible toxicity similar to plasmid-based transfection (Fig. 3d).

Baculovirus transduction of mammalian cells is transient in nature as the foreign DNA does not integrate into the host genome. We therefore tested the persistence of recombinant expression following transduction with a MultiPrime baculovirus by immunofluorescence and western blotting. In our experiments, the percentage of positive cells decreased to ∼20% after 20 days and 5% after 30 days (Fig. 3e).

### Modulation of expression levels in mammalian cells

**Figure 4:**
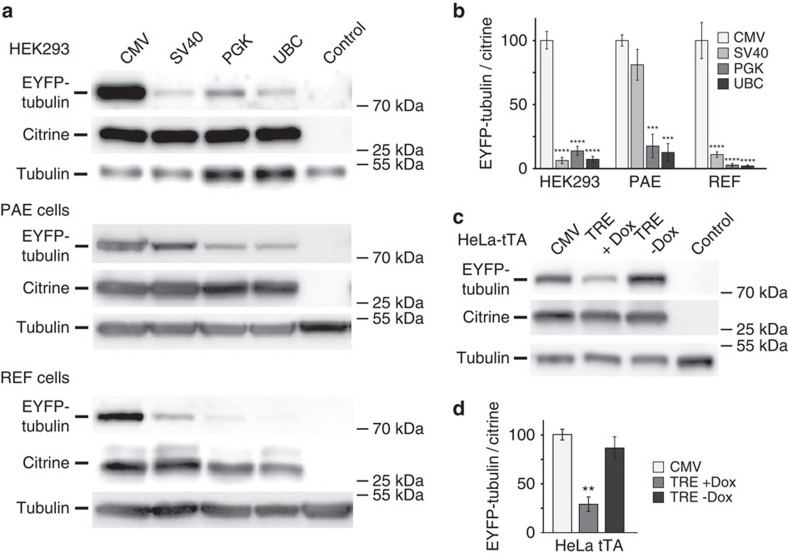
Modulation of expression levels with alternative promoters. (**a**) Transduction of HEK, PAE and REF cells with baculoviruses expressing EYFP-tubulin under the control of the indicated promoters and citrine under the control of the CMV promoter is shown. (**b**) Quantification of the blots. EYFP-tubulin/citrine ratio was used as a measure for promoter strength. Endogenous tubulin was used as loading control. Data shows mean value±s.d.; *n*=3; ****P<0.0001, ***P<0.001 determined by comparing CMV promoter with alternative promoters using one-way analysis of variance followed by the Dunnet's *post hoc* test. The CMV promoter is the strongest promoter in all tested cell lines. (**c**) HeLa-tTA cells were infected with a baculovirus expressing EYFP-tubulin under the control of a tetracyclin inducible element and citrine under the control of the CMV promoter. (**d**) Quantification of the blots shown above. Data shows mean value±s.d.; *n*=3; **P<0.01 determined by comparing induced versus non-induced tet promoter using one-way analysis of variance followed by the Tukey *post hoc* test. No significant difference between CMV promoter and induced tet promoter.

The hCMV-IE1 promoter we used in our experiments is considered to be the strongest promoter available for heterologous expression in most mammalian cells. It may be desirable to have alternative promoters that are characterized by lower levels of expression. We expanded our tool-box by incorporating the SV40, PGK and UBC promoters in alternative expression cassettes in our MultiPrime system (Supplementary Fig. 6). We determined expression levels from these alternative promoters by expressing EYFP-tubulin, and simultaneously expressing citrine from a CMV promoter as a bench-mark to normalize expression levels. All three alternative promoters show distinctly lower expression levels in HEK293 and PAE cells as well as in primary REF compared to CMV promoter-driven expression (Fig. 4a,b). Furthermore, we included a tetracycline-inducible promoter in our system (Supplementary Fig. 6). Tetracycline-inducible promoters are dependent on a transactivator, for example tTA, to initiate expression^29^. We observed approximately four times higher expression levels in the absence of doxycycline in HeLa cells stably producing tTA, which were transduced with a MultiPrime baculovirus containing a tetracycline-inducible expression cassette, in good agreement with reports involving tetracycline-inducible promoters on plasmids (Fig. 4c,d).

### Bifunctional dual-host promoters

**Figure 5:**
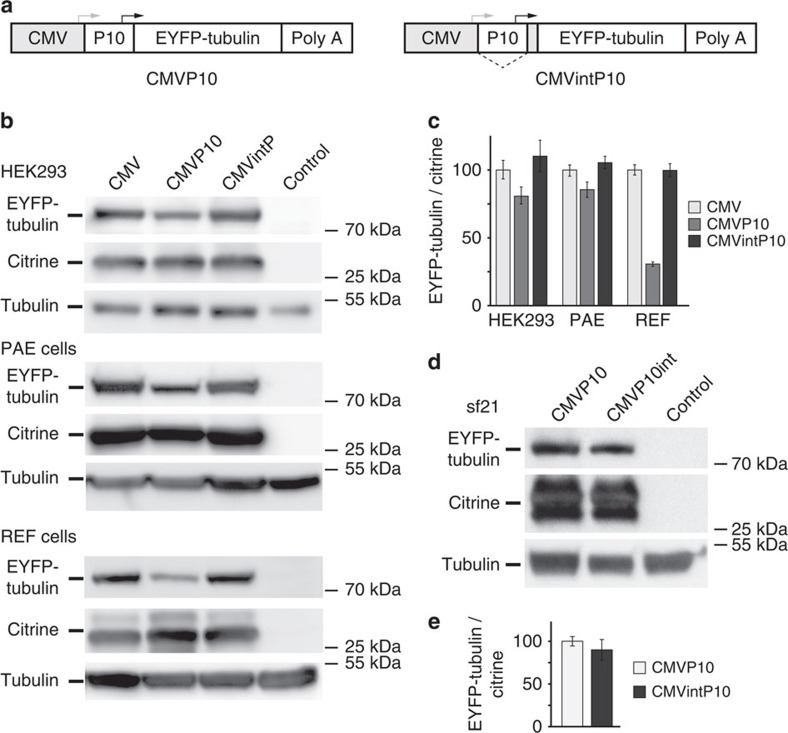
Promoters active in mammalian and insect cells. (**a**) Structure of the tested dual promoters is shown schematically. In CMVP10, the baculoviral very late promoter p10 was inserted downstream of the CMV promoter. In CMVintP10, the p10 promoter was placed within an intron and is spliced out from the transcript of the CMV promoter. Grey arrow: transcription initiation of the CMV promoter; black arrow: transcription initiation of the p10 promoter. (**b**) HEK293, PAE and REF cells were infected with a MultiPrime baculovirus expressing EYFP-tubulin under the control of the above promoters and citrine under the control of the CMV promoter. The lysates of the cells were analysed by western blotting. (**c**) Quantification of blots shown in **b**. EYFP-tubulin/citrine ratio was used as a measure for promoter strength. Endogenous tubulin was used as loading control. The CMVintP10 promoter expresses at a similar level as the original CMV promoter, while the CMVP10 promoter expresses at a lower level in mammalian cells. (**d**,**e**) The same baculoviruses were used to infect insect (Sf21) cells. Citrine was expressed by a P10 driven expression cassette in the backbone of the baculovirus. Data shows mean value±s.d.; *n*=3; there is no significant difference (*P*>0.05) by comparing the two CMVP10 promoter variants using the Student's *t*-test.

Expression plasmids that could be used for heterologous protein production in insect as well as in mammalian cells have not found wide-spread application so far, possibly because comprehensive comparative data which would have encouraged their use is currently lacking. We addressed this issue by creating, validating and incorporating dual-host promoters as a choice in our MultiPrime system. Our objective was to provide a single expression reagent, which is the composite MultiPrime baculovirus containing the genes of choice controlled by this validated dual-host promoter, for example to produce a protein or protein complex of choice efficiently in insect cells for structural studies and in mammalian cell lines for functional validation. We used two promoters, the first one (denoted CMVP10) is a fusion of the CMV promoter and the baculoviral very late promoter p10, the second (denoted CMVintP10) contains the p10 promoter in an intron of the CMV transcription unit (Fig. 5a and Supplementary Fig. 7). These two dual-host promoters were validated in mammalian cells by expressing EYFP-tubulin from a MultiPrime baculovirus, which also expressed citrine driven by a CMV promoter for normalization purposes. In HEK293 and PAE cells, the dual function promoters expressed at comparable levels to the original mammalian-only CMV promoter. In REF cells, the intron-less CMVP10 promoter resulted in lower expression (Fig. 5b,c). We quantified expression from these MultiPrime baculoviruses in insect cells and found them entirely satisfactory (Fig. 5d,e). Furthermore, we tested MultiPrime constructs expressing human transcription factors, which we had produced before for structural studies in insect cells with our MultiBac insect-cell expression system (Supplementary Fig. 8)^30^,^31^. We observed virtually indistinguishable levels of expression for complexes formed by these human TATA-box associated factors (TAFs) from dual function promoters as compared to the MultiBac expressed complexes. Transduction of HeLa cells with the TAF producing MultiPrime baculoviruses resulted in close to complete transduction rates (Supplementary Fig. 8).

### Genome engineering by CRISPR/Cas9

**Figure 6:**
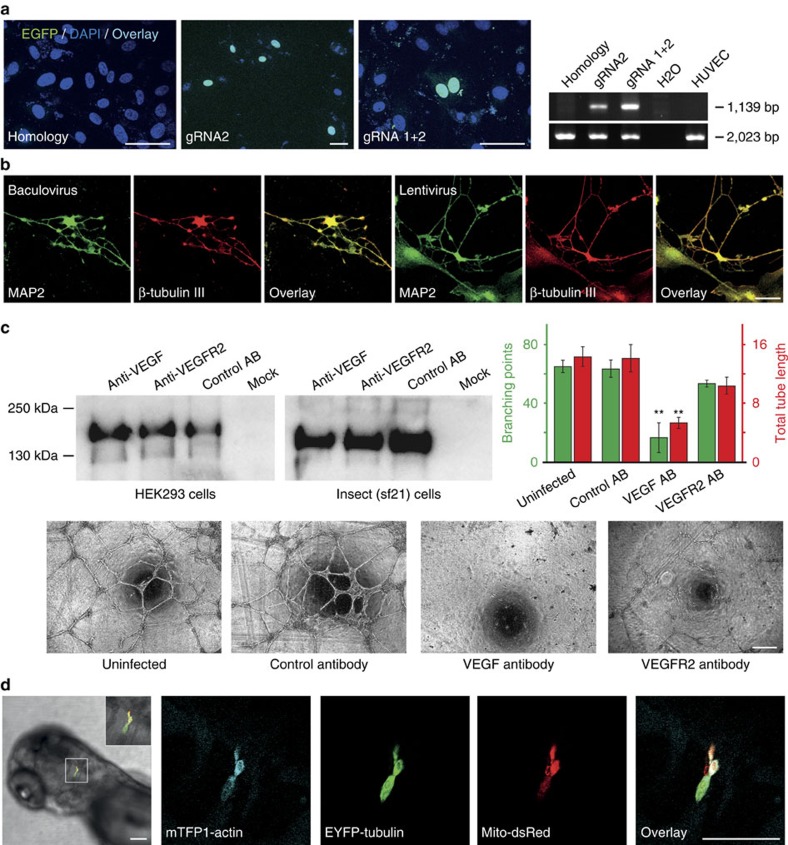
MultiPrime applications. (**a**) Genome engineering. Infection of HUVEC with a baculovirus containing a HMGA1-EGFP homology construct did not lead to cells with HMGA1-EGFP expression (left). Co-expression with Cas9 and HMGA1-gRNA1 (2nd panel) or co-expression with Cas9, HMGA1-gRNA1 and HMGA1-gRNA2 (3rd panel) led to cells with HMGA1-EGFP expression in the nucleus. Correct integration of the homology construct was verified with PCR. The wild-type allele yielded a fragment of 2,023 bp, whereas the mutant allele results in a fragment with 1,139 bp (right). Scale bar, 50 μm. (**b**) Reprogramming of cells. MEF cells were infected with a Multiprime virus expressing Ascl1, Brn2 and Myt1L or co-infected with three lentiviruses individually expressing the same transcription factors. Both strategies led to cells with neuron-like morphology that express the neuronal markers MAP2 and β-tubulin III. Scale bar, 50 μm. (**c**) Functional antibody expression. MultiPrime viruses, encoding light and heavy chains of three different IgGs (anti-VEGF, anti-VEGFR2, and unspecific) were used to express antibodies in HEK293 (left) and also in insect cells (middle). HUVEC cells were infected with these viruses and the cells were used in a Matrigel-based angiogenesis assay. As expected, only the anti-VEGF antibody is capable of blocking tube formation Scale bar, 500 μm. Data shows mean value±s.d.; *n*=4; **P<0.01 when comparing VEGF function-blocking antibody versus control antibody. There is no significant difference (*P*>0.05) when comparing the non-function-blocking VEGFR2 antibody with the control antibody. Both *P* values are determined using one way analysis of variance followed by the Dunnet's *post hoc* test. (**d**) Baculovirus-mediated gene expression in zebrafish. Dorsal view of the head of a 3-day-old zebrafish larva after injection of MultiPrime baculoviruses expressing mTFP1-actin, EYFP-tubulin and Mito-DsRed into the hindbrain region at 24 h post fertilization. All infected cells express all heterologous proteins. Scale bar, 100 μm.

CRISPR/Cas9-mediated genome engineering requires the expression of Cas9, the concomitant expression of a U6-driven guide RNA (gRNA) and the provision of a DNA construct for homologous recombination. Currently used viral systems can harbour Cas9 and the gRNA but are unable to include a homology construct due to limited cargo capacity^32^. We assembled DNAs for the expression of a HMGA1-EGFP fusion protein from its endogenous locus in a MultiPrime virus^33^ (see Supplementary Fig. 9 for details). Transduction of HEK293 and HUVEC led to expression of HMGA1-EGFP in the nucleus in ∼1% of the cells. Successful homologous integration of the DNA construct was verified by PCR (Fig. 6a and Supplementary Fig. 9).

### Reprogramming by MultiPrime

We next investigated whether MultiPrime viruses are suitable for reprogramming of cells. Currently, this is mainly carried out with lentivirus, which is a retrovirus that stably integrates into the genome of cells. We assembled a MultiPrime virus expressing the transcription factors Asc1, Brn2 and Myt1L, which were shown to convert MEFs into neurons^7^. Transduction of MEFs with this MultiPrime virus resulted in cells with neuron-like morphology, which expressed the neuronal markers MAP2 and β-tubulin III 20 days after transduction, indistinguishable from co-infection with three lentiviruses each expressing one of the transcription factors (Fig. 6b). Our results provide compelling evidence that reprogramming can be successfully achieved with a transient expression system such as MultiPrime.

### Functional antibody production

Our MultiPrime approach can not only induce morphological changes in cells but also potentially interfere with it. We addressed this by using MultiPrime to express functional antibodies in primary cells. Our previously described single-chain antibodies targeting VEGF (SZH9) and VEGFR2 (ADH9), and a control single-chain antibody (A1) were converted into a full length IgG consisting of light and heavy chains^34^,^35^. The dual-host promoter CMV-CMVintP10 was utilized to drive recombinant IgG expression. The resulting MultiPrime baculovirus was successfully tested for expression in HEK293 and insect cells (Fig. 6c). All IgG antibodies tested were expressed at comparable levels. The same virus was then used to transduce HUVEC that were then placed into a tube formation assay. Only the function-blocking anti-VEGF antibody SZH9 was able to interfere with tube formation. All other antibodies, including the VEGFR2-binding but not function-blocking antibody ADH9, did not interfere with tube formation (Fig. 6c).

### Zebrafish transduction

It was previously shown that mammalian promoters can be used for heterologous expression in zebrafish embryos^36^. We set out to establish whether MultiPrime viruses are restricted to mammalian and insect cells, or whether they can also be used to transduce zebrafish. A MultiPrime virus encoding mTFP1-actin, EYFP-tubulin and Mito-dsRed under control of mammalian CMV promoters was injected into intercellular spaces in the brain region of zebrafish embryos at 24 h post fertilization. Injection of this virus showed heterologous expression of all genes in zebrafish embryos. Expression was restricted to the site of injection and could be detected for at least 5 days (Fig. 6d).

## Discussion

In the three decades since their inception, baculovirus-based expression systems have become well-established and widely used for recombinant protein production in insect cells. Later, it was discovered that baculoviruses not only infect insect cells but can also drive heterologous protein expression in mammalian cells if appropriate mammalian regulatory elements are provided in the recombinant baculovirus genome^22^,^23^,^37^. This so-called ‘BacMam' method has been applied to produce heterologous proteins in academic and industrial research and development, notably for pharmacological screening^37^. Today, it is becoming increasingly evident that most physiological activities are mediated by multiple proteins forming complex assemblies. Therefore, a powerful tool that exploits recombinant baculovirus to deliver multiple genes simultaneously and reproducibly into a range of mammalian cell types and notably primary cells is highly desirable to study health and disease states, and to analyze molecular mechanisms of cell fate. Notwithstanding, such a tool has been lacking so far. Therefore, we developed MultiPrime, a versatile, flexible and fully modular system for efficient multigene delivery and expression in any mammalian cell type, primary and established. MultiPrime relies on a set of customized DNA plasmid modules, called acceptors and donors that provide the means to combine a theoretically unlimited number of genes of interest with different promoters, terminators and other control elements in multiple expression cassettes to generate multigene-delivery constructs, which are then inserted into engineered baculoviral genomes. Moreover, they can comprise all the elements necessary for genome engineering including editing functions and the sequences required for homologous recombination. We provide and validate a range of mammalian promoters that can be introduced into our MultiPrime system in this way. In addition, we provide dual-host promoters to drive heterologous multiprotein production in both insect and mammalian cells. This highly versatile and flexible tool-box allows users to conveniently introduce many different proteins simultaneously into mammalian and insect cells. Corroborating previous observations, we found negligible toxicity and sustained viability when infecting a range of mammalian cells with recombinant MultiPrime reagents. Importantly, we demonstrate here that MultiPrime infected cells are competent to divide and migrate normally and are capable of adequately responding to external stimuli as, for example, growth factors.

In this study, we utilized two baculovirus types, EMBacY and MultiBacMam. These engineered baculoviral genomes are characterized by reduced proteolysis and delayed cell lysis during virus amplification in insect cells, resulting in high quality, high titre virus^25^. The EMBacY and MultiBacMam viruses express either EYFP or mCherry fluorescent marker genes, to signal late replication cycle entry. Expression of fluorescent marker proteins during virus production is a convenient tool to simplify and standardize monitoring the production of baculovirus, in particular for non-specialist users. MultiBacMam virus generated in this study expresses also VSV-G during virus production in insect cells. Decorating baculovirus with VSV-G has been shown to improve mammalian transduction efficiencies. Consequently, we observed superior transduction efficiencies with our MultiBacMam-derived viruses that display VSV-G on their capsids. We note here that, at least in Switzerland where these experiments were performed, MultiBacMam-derived reagents expressing VSV-G have to be handled at biosafety level 2, which requires specific laboratory infrastructure. To circumvent this complication the EMBacY virus variant can be utilized, which is devoid of VSV-G but still resulted in satisfactory transduction rates in our experiments. Nonetheless, for experiments which may rely on maximum transduction efficiencies, the VSV-G containing MultiBacMam virus is recommended.

All viral genomes we utilized contain a site-specific integration site in the backbone distal from the Tn7-attachment site. This LoxP site allows introduction of additional genes by Cre-LoxP mediated fusion *in vivo*^12^. This enables a range of options to modify and tailor the baculovirus genomes for specific applications. For example, a baculovirus called SweetBac was developed to achieve mammalian-type glycosylation of recombinant secreted proteins such as antibodies^38^,^39^. Currently, these functionalizations are limited to applications in insect cells. We anticipate that a wide range of functions to modify, enhance and regulate multiprotein production in mammalian cells will be exploited by modifying the baculoviral genome accordingly, providing appropriate expression cassettes active in mammalian cells in the LoxP locus of these vectors.

Multigene expression systems are rapidly gaining prominence for producing protein complexes for structural and functional studies. Often, several expression systems must be tested to obtain functional complexes in sufficient quantity and quality. This typically requires recloning of genes into different sets of expression plasmids given that the regulatory elements in each system, here mammalian and insect cells, are optimized for a particular host, and are typically not compatible between the different species. The incorporation of dual-host promoters into MultiPrime allows simultaneous testing of expression constructs in insect and mammalian cells by using the same reagent. This feature can be conveniently exploited if high-level production of a complex protein of interest is carried out in insect cells, while functional analysis of the same complex is performed in mammalian cells, which is increasingly the case in current structural biology. The possibility to use the same reagent for both host systems will also benefit analysis of structure–function correlations requiring multiple mutational analysis. MultiPrime affords the means to carry out such elaborate studies, notably also of complexes controlling cell fate, which can be mechanistically dissected by infecting primary cells.

Baculovirus constitutes an attractive tool for gene therapy for a number of reasons. Due to its flexible envelope structure, very large heterologous DNA cargo can be incorporated into the baculoviral genome. Moreover, baculovirus is replication incompetent in mammalian cells, and virtually no viral protein expression occurs on transfection in a mammalian host. Initial *in vivo* experiments had limited success since injected baculoviruses are rapidly inactivated and cleared by the immune complement system. Strategies were developed to overcome this impediment and many successful *in vivo* applications were published since then (reviewed in ref. 24). For example, expression of VEGF-D-induced vascularization in rabbit skeletal muscle suggesting that baculovirus-driven VEGF-D expression might be an option to cure lymphatic disorders^40^. Nevertheless as a non-integrative virus it is *a priori* limited to transient expression, which can be an advantage or a disadvantage depending on the application. Transient expression may be desirable, for example, for vaccination or to promote changes in cell fate. Of note, altering cell fate is a particularly interesting application for multigene expression systems, as it relies on the simultaneous and temporally restricted expression of several transcription factors. Induced-pluripotent stem cells have been generated before with a BacMam virus *in vitro* using a fusion protein construct^41^. Four transcription factors were expressed as a fusion protein from a single open reading frame (ORF) via self-cleaving 2A peptides. Here, we converted MEFs into neurons using independent expression cassettes, which offer advantages especially when different protein combinations need to be tested in a combinatorial fashion.

MultiPrime is not restricted to the delivery of expression constructs. We anticipate that genome engineering will be an important future application, owed to the very large cargo capacity of baculoviruses. Other viruses such as lentiviruses or adeno-associated viruses cannot accommodate all DNA elements needed to produce Cas9, a gRNA and a construct for homologous recombination. With MultiPrime, we were able to modify the HMGA1 locus of HUVEC, which are human primary cells that show restricted replication potential.

Our results compellingly validate MultiPrime as a powerful vehicle for multigene delivery, protein expression and genome engineering, relevant for a large number of applications, *in vitro* and *in vivo*, and underscore the enormous potential of our baculoviral system to deliver large multigene DNA constructs into a wide range of mammalian cells, notably including primary cells. A multitude of genes and regulatory elements can be delivered due to the very large heterologous DNA cargo capacity of the system, offering novel exciting possibilities for biological research. Entire signalling cascades, gene regulatory systems or metabolic pathways and multiple mutants thereof, can be efficiently engineered with MultiPrime. We anticipate that many applications will benefit from MultiPrime, notably when efficient transfer of multiple genes or efficient engineering of genomes is required.

## Methods

### Molecular biology

DNA construction in MultiPrime follows the high-throughput compatible logic of our ACEMBL concept to prepare multicomponent DNA constructs from acceptor and donor plasmid DNA modules that are conjoined by the Cre-LoxP fusion reaction^13^,^14^. Plasmid pSI-AGR10 is the common acceptor in MultiPrime, and has been developed from our previous pFL plasmid^25^. The ampicillin resistance gene and an internal SapI site were removed and the expression cassettes for insect-cell expression replaced by the CMV-based expression cassette from plasmid pSI-AKR1 by standard cloning methods (Supplementary Fig. 2a)^14^. In addition, pSI-AGR10 contains a gentamycin resistance marker, a LoxP site, and the DNA elements (Tn7R, Tn7L) required for transposition into the baculovirus genome by Tn7 transposase.

All donor plasmids of the original MultiLabel system are compatible with this acceptor^14^. Donors are fused to pSI-AGR10 by Cre-LoxP recombination concomitantly or in a sequential manner. Acceptor–donor assembly was performed as described and electrocompetent DH10β or CaCl_2_ competent XL1-blue cells were used for transformation^42^. Sequences were assembled *in silico* using the ‘Multi-Cre Recombination Tool' in the plasmid editor software Ape (http://biologylabs.utah.edu/jorgensen/wayned/ape/) or, alternatively, with software Cre-ACEMBLER^43^. Integrity of all fusion plasmids was confirmed by restriction mapping. Alternative mammalian promoters and dual-host promoters active in both mammalian and insect cells were synthesized by Genewiz (South Plainfield, USA) on the basis of sequences provided in the Supplementary Materials and inserted as AscI–HindIII fragments into parent Acceptor plasmid pSI-AGR10.

### Recombinant baculoviral genomes

Two baculoviral genomes were used in this study, our previously described EMBacY genome and the novel MultiBacMam genome, which we constructed in this study. Both baculoviral genomes are present as bacterial artificial chromosomes (BAC) in *E. coli* cells (DH10EMBacY and DH10MultiBacMam, respectively). EMBacY produces yellow fluorescent protein (YFP) as a marker in infected insect cells as a means to track virus amplification and performance by monitoring the fluorescence signal^25^.

Display of a VSV-G on the baculovirion was reported to enhance mammalian transduction efficiency by baculovirus^27^. We therefore constructed a novel MultiBacMam baculovirus by modifying our original MultiBac baculoviral genome, retaining its advantageous features including reduced proteolysis and delayed cell lysis^12^. A synthetic gene (Genscript, Psicataway, NJ) encoding for VSV-G was inserted into a modified pUCDM donor plasmid^25^ by using BamHI and XbaI restriction sites to yield plasmid pLox-VSV-G. Subsequently, a second cassette containing a synthetic gene for mCherry (Genscript) was inserted by using the multiplication module as described^13^. The resulting pLox-VSV-G-mCherry Donor plasmid was incorporated into the MultiBac virus by transforming DH10MultiBac^Cre^ cells harbouring the MultiBac baculoviral genome as a BAC and Cre recombinase expressed from a pBADZHisCre helper plasmid on arabinose induction^12^. Positive integrands were selected by antibiotic screening. Successful Cre-mediated integration was further verified by PCR analysis as described^44^. Competent DH10MultiBacMam cells were prepared following standard protocols and contain in addition to the MultiBacMam baculovirus also a helper plasmid expressing Tn7 transposase on induction with isopropyl β-D-1-thiogalactopyranoside. Expression of mCherry from this baculovirus in infected insect cells during virion production results in the cell culture adopting a characteristic purple color, allowing tracking of successful viral infection and production easily by eye.

### Generation of composite MultiPrime baculovirus

MultiPrime acceptors or acceptor–donor fusions were transformed into electrocompetent DH10EMBacY or DH10MultiBacMam cells, respectively. Composite baculovirus generation occurred by Tn7 tranposition mediated by Tn7 transposase expressed from a helper plasmid. Transformants were selected and composite baculoviral genomes prepared as described^44^ Sf21 insect cells were transfected with Cellfectin II (Life Technologies) at a density of 0.5 × 10^6^ cells ml^−1^ according to manufacturer's recommendations. We took particular care during virus amplification to prevent accumulation of defective virus, which would not express all heterologous genes. We applied a protocol we had developed previously for successful amplification of composite baculovirus containing multiple copies of viral late promoters (polh, p10), preserving the integrity of the viral genome^28^. Briefly, primary baculovirus stock (V0, 2 ml) was harvested 50–60 h after transfection and 0.5 ml was used to infect 4 ml new Sf21 cells for 75 h yielding V1 stock. Overall 3 ml of this V1 baculovirus stock was then used for a further round of virus amplification for 60 h (V2, 100 ml). The amplification of the virus was followed in this phase by monitoring EYFP (EMBacY) or mCherry (MultibacMam) expression from the viral backbone (Supplementary Fig. 1). Less than 1% of cells were positive when harvesting V0. When harvesting V1, 20–30% of cells were positive and after V2, 80–90% of cells were positive. Incubation times must not be extended during amplification, otherwise over-amplification of the virus can occur, resulting in loss of heterologous insert (Supplementary Fig. 4). The V2 virus stocks were stored either at 4 °C or after addition of 5% FBS at −80 °C. For sensitive cells (for example, iPS) or zebrafish, virus was concentrated by ultracentrifugation. For this purpose, virus supernatant was placed on a sucrose cushion (25% sucrose/ 5 mM NaCl/ 10 mM EDTA) and then centrifuged for 90 min at 80,000*g*. The pellet containing the virus was resuspended in PBS pH 6.2 (ref. 45). The titre of baculovirus stocks was determined using end-point dilution assay^46^. Viruses displaying VSV-G were handled as biosafety level 2 agents in Switzerland.

### Cell culture

Insect cells (Sf21, Sf9; Life Technologies) were cultured in SF-4 BaculoExpress ICM medium (Amimed) containing 1% FBS at 27 °C. Mammalian cells were incubated at 37 °C in a humidified atmosphere containing 5% CO_2_. HEK293, COS7, REF, Swiss 3T3 and HeLa cells were cultured in DMEM (Amimed) containing 10% FBS (Life Technologies) and 100 units ml^−1^ penicillin and 100 μg ml^−1^ Streptomycin (Life Technologies). PAE and HUVEC (Life Technologies) cells were maintained in Ham-F12 media supplemented with 10% FBS and penicillin/ streptomycin and M-200 medium (Life Technologies), respectively. Primary rat cortex neurons (Life Technologies) were cultured in Neurobasal medium supplemented with 1% B27 and 1% Glutamax (Life Technologies). Human iPS cells (NAS2) were obtained from Tilo Kunth (University of Edinburgh) and cultured in feeder-free maintenance medium for human ES/ iPS cells (mTESR1 medium; Stem Cell Technology)^47^. iPS cells were immunostained with an Oct4 antibody (Santa Cruz Biotechnology, sc-5279, 1:500 in 10% NDS/ 0.2% Triton X100/ 1% BST/ PBS), transfections were performed with FusionHD (Promega) according to manufacturer's recommendations and cells were analyzed 42 h after transfection. For endothelial tube formation assays, MultiPrime-transduced HUVEC cells were seeded on a ibiTreat μ-slide angiogenesis plate (ibidi GmbH, Germany) at a density of 5,000 cells/slide in EGM-2 medium (Life Technologies) and analyzed 16 h later. Migration assays were performed by seeding 21000 MultiPrime-transduced HUVEC cells in ibidi Culture-Insert plate (ibidi GmbH, Germany). The culture-insert was removed after 14 h and migration into the gap was monitored every 2 h for 24 h.

### Transduction of mammalian cells

Mammalian cells were plated at a density of 2.5 × 10^5^ cells per well in six-well plates 1 day before transduction. Baculovirus was added at a MOI between 100 and 500 in 80% insect medium/ 20% DMEM without any FBS or antibiotics. Transduced cells were incubated at 37 °C for 8 h, and the medium was then replaced with fresh mammalian cell culture medium. Plates were cultured for one or two additional days. Cytotoxicity of baculovirus transduction was monitored by MTT assay in various mammalian cells lines. Overall 10^4^ cells were plated in a 96-well plate and transduced with baculovirus at a MOI 500. After 24 h, the medium was replaced with culture medium containing 20 μM resazurin and the cells were incubated for 2–4 h. The number of viable cells was obtained by monitoring resazurin fluorescence with a microplate spectrofluorometer (Tecan Ltd).

For CRISPR/Cas9-mediated genome engineering, HEK293 cells and HUVEC were transduced with a Multiprime virus that expresses CMV-driven Cas9, U6-driven gRNAs and a homology construct as described in Supplementary Fig. 9. Immunostaining for Cas9 after 40 h revealed 80% transduction. Cells were fixed after 4 or 6 days and analyzed for nuclear EGFP expression by microscopy or DNA was extracted with QIAamp DNA mini kit (Qiagen). Correct integration was verified by PCR using primers described in Supplementary Fig. 9.

MEF cells used for reprogramming to neurons were obtained from Amsbio, and were used at passage 3. Cells were transduced with concentrated bacuolovirus expressing Ascl1, Brn2 and Myt1L in MEF medium for 8 h. In parallel, MEFs were infected with lentiviruses containing expression constructs Tet-o-FUW-Ascl1, Tet-o-FUW-Brn2 and Tet-o-FUW-Myt1L (all from Addgene) in presence of polybrene (8 μg ml^−1^). Cells were cultured in N3 medium (DMEM/F12, B27, N2 (all (Life Technologies), 25 μg ml^−1^ Insulin (Sigma-Aldrich)). Doxycycline (2 μg ml^−1^) was added to lentivirus-transduced cells^7^,^48^. Cells were fixed after 6, 12 and 20 days. Immunostaining was performed with chicken anti MAP2 (Neuromics, CH22103, 1:5,000 in 10% NDS/ 0.2% Triton X100/ 1% BST/ PBS) and mouse anti β-tubulin III (Sigma, T8578, 1:600 in 10% NDS/ 0.2% Triton X100/ 1% BST/ PBS) antibodies.

### Microscopy

Cells for microscopic analysis were plated on glass coverslips. Untreated coverslips were used for COS7, REF, Swiss 3T3 and PAE cells. Poly-L-lysine (Sigma P4707) treated coverslips were used for HEK293 cells and 0.1% gelatin (Sigma G1393) treated coverslips for HUVEC. Poly-D-lysine hydrobromide coated coverslips (Sigma P7280) were used for primary rat cortex neurons and iPS were plated on hESC qualified Matrigel (BD Bioscence). Analysis of cells was performed 27, 42 and 48 h after transduction. Cells were fixed with 4% formaldehyde in PBS and mounted with Gelvatol. Imaging was performed on a Leica SP5 laser scanning confocal microscope or on an Olympus IX81 equipped with an Andor iXonEM camera. On Leica SP5, EBFP2 was excited with the 405 nm laser line and the emission was collected from 430 to 450 nm (405/ 430-450). The other fluorescent proteins were analyzed as follows: mTFP1 (458/ 485-510), mCitrine (514/ 525-545), mCherry (543/ 585-620) and mPLUM (633/ 640-800). In addition, the spectral mode (xyλ) of the microscope was used to verify the presence of all fluorescent proteins (data not shown). Standard excitation and emission filters were used on the Olympus IX81. Quantification was performed with Squassh^49^.

### Western blotting

Mammalian cells were lysed 42 h after transduction with lysis buffer (0.5% Triton X100, 50 mM Tris–HCl, 100 mM NaCl, pH 7.5). The supernatant was used for western blotting after sonification and centrifugation. Rabbit anti-GFP (Abcam ab137827; diluted 1:2500 in 3% BSA/TBST) and mouse anti-tubulin (Sigma T5168; diluted 1:2,500 in 3% BSA/TBST) were used as primary antibodies. As secondary antibodies, alkaline phosphatase-coupled goat anti-rabbit and anti-mouse as well as donkey anti-human IgGs (Southern Biotech, diluted 1:10,000 in TBST) were used, followed by chemiluminescence detection. Quantification was performed with ImageJ. Original western blots are shown in Supplementary Fig. 10.

### Transduction of zebrafish embryos

All experiments were performed in accordance with the animal welfare guidelines of the Federal Veterinary Office of Switzerland. Zebrafish (*Danio rerio*) were maintained as described^50^. Embryos of the wild-type strain WIK were raised at 28°C in E3 medium (5 mM NaCl, 0.17 mM KCl, 0.33 mM CaCl_2_ and 0.33 mM MgSO_4_), and pigment development was inhibited by phenylthiourea (1-phenyl-2-thiourea; Sigma-Aldrich) as described in Westerfield^50^. For injections, individual dechorionated embryos at 24 h post fertilization were anesthetized in 200 mg ml^−1^ 3-aminobenzoic acid methyl ester (MESAB, Sigma-Aldrich) and 4.6 mM NaHCO_3_ and embedded in 1% low melting agarose (Lonza). Concentrated viruses were injected in intercellular spaces using borosilicate glass microcapillary injection needles (1 mm outside diameter × 0.78 mm inside diameter, Science Products GmbH, Hofheim, Germany) and a PV820 Pneumatic PicoPump (World Precision Instruments, Sarasota, Florida, USA). After injection, the infected embryos were returned to E3 medium. For in vivo imaging, the larvae were anesthetized and embedded in 1% low melting agarose in a 35 mm imaging dish with a glass bottom (Ibidi) and imaged using a CLSM SP5 Mid UV–vis Leica inverted microscope.

### Bifunctional CMV/ P10 dual-host promoters

Subcomplexes of human general transcription factor TFIID were produced in insect cells from the CMV-CMVintP10 promoter (Supplementary Fig. 7). A complex formed by human TBP associated factor (TAF) 8 and TAF10, and a complex formed by TAF5, TAF6 and TAF9, were expressed. Production levels of these complexes on the basis of the dual-host promoter was compared to previously established production levels on the basis of the baculoviral polyhedrin (polh) promoter. A polyprotein strategy was utilized^51^, which we had developed for high-level expression of complexes^30^,^31^. Briefly, the genes encoding for the TAFs were placed in a single ORF flanked by genes encoding for tobacco etch virus NIa (TEV) protease at 5′ and a cyan fluorescent protein at the 3′-end^51^. The ORFs give rise to self-processing polyproteins, which are cleaved by TEV protease at high specific TEV protease cleavage sites in between the constituent proteins. The polyproteins are shown schematically in Supplementary Fig. 8.

Polyproteins were expressed from the CMVintP10 and the polh promoter, respectively, by using EMBacY, and purified as described^30^,^31^. Production levels of the polyproteins in insect cells were indistinguishable notwithstanding the promoter used (Supplementary Fig. 8). Next, the MultiBacMam virus (see above) was used in conjunction with the CMVintP10 promoter, again leading to indistinguishable expression levels (Supplementary Fig. 8). HeLa cell cultures were transfected with MultiBacMam-derived baculoviruses that had been used to express the proteins in insect cells. Complete DMEM containing 10% foetal calf serum and 8 mM L-glutamine was utilized. Best results were achieved by supplementing the media with 3 mM sodium butyrate for cell recovery after aspirating the virus. Nearly all HeLa cells were transduced as revealed by measuring the specific fluorescence of the cyan fluorescent protein marker encoded by the polyprotein (Supplementary Fig. 8).

## Additional information

**Accession codes**: Nucleotide sequences have been deposited in GenBank under the following accession codes: pSI-AGR10: KX001770; pSI-AGZ10: KX001771; pSI-DSZ2cx-TRE: KX001772; pSI-DSZ2cx-DI: KX001773; pSI-DSZ2x-DM: KX001774; pSI-DSZ2cx-PGK: KX001775; pSI-DSZ2cx-UBC: KX001776; pSI-DSZcx-SV40: KX001777; pSI-AGL10: KX001778; pSI-AGL10-DM: KX001779; pSI-AGL10-DI: KX001780; pSI-DAZ-DM: KX001781; pSI-DAZ-DI: KX001782.

**How to cite this article**: Mansouri, M. *et al*. Highly efficient baculovirus-mediated multigene delivery in primary cells. *Nat. Commun.* 7:11529 doi: 10.1038/ncomms11529 (2016).

## Supplementary Material

Supplementary Information Supplementary Figures 1-10

Supplementary Movie 1 EGF uptake of COS7 cells. COS7 cells that endogenously express EGFR were transduced with a virus that expresses mTFP1-RAB7, mCitrine-RAB5, and mCherry-RAB11A. Cells were stimulated with Cy5-labelled EGF forty hours after transduction and then monitored for 180 minutes (left panel). The middle panel shows a higher magnification before (top) and after segmentation with Squassh (bottom). The right panel shows quantification of colocalization of EGF with the different RAB GTPases. As expected, EGF colocalizes first with RAB5 and then with RAB7. Little colocalization with RAB11A was observed.

Supplementary Movie 2 VEGF A165a uptake of PAE cells stably expressing VEGFR2 and NRP1. PAE cells were transduced with a virus that expresses mTFP1-RAB7, mCitrine-RAB5, and mCherry-RAB11A. Cells were stimulated with NT647-labelled VEGF A165a forty hours after transduction and then monitored for 150 minutes (left panel). On top, the original movie is shown. The Squassh segmented movie is shown below. The right panel shows quantification of colocalization of VEGF A165a with the different RAB GTPases. VEGF A165a colocalizes with RAB5, RAB7, and RAB11A. This is in line with previous observations that VEGFR2 can be recycled or degraded when bound to VEGF A165a.

Peer review file
