# Supplementary material for: Highly efficient baculovirus-mediated multigene delivery in primary cells
Source: Nat Commun. 2016 May 4;7:11529. doi: 10.1038/ncomms11529 (PMC4857464; doi:10.1038/ncomms11529)

Supplementary Figure 1

Workflow of MultiPrime

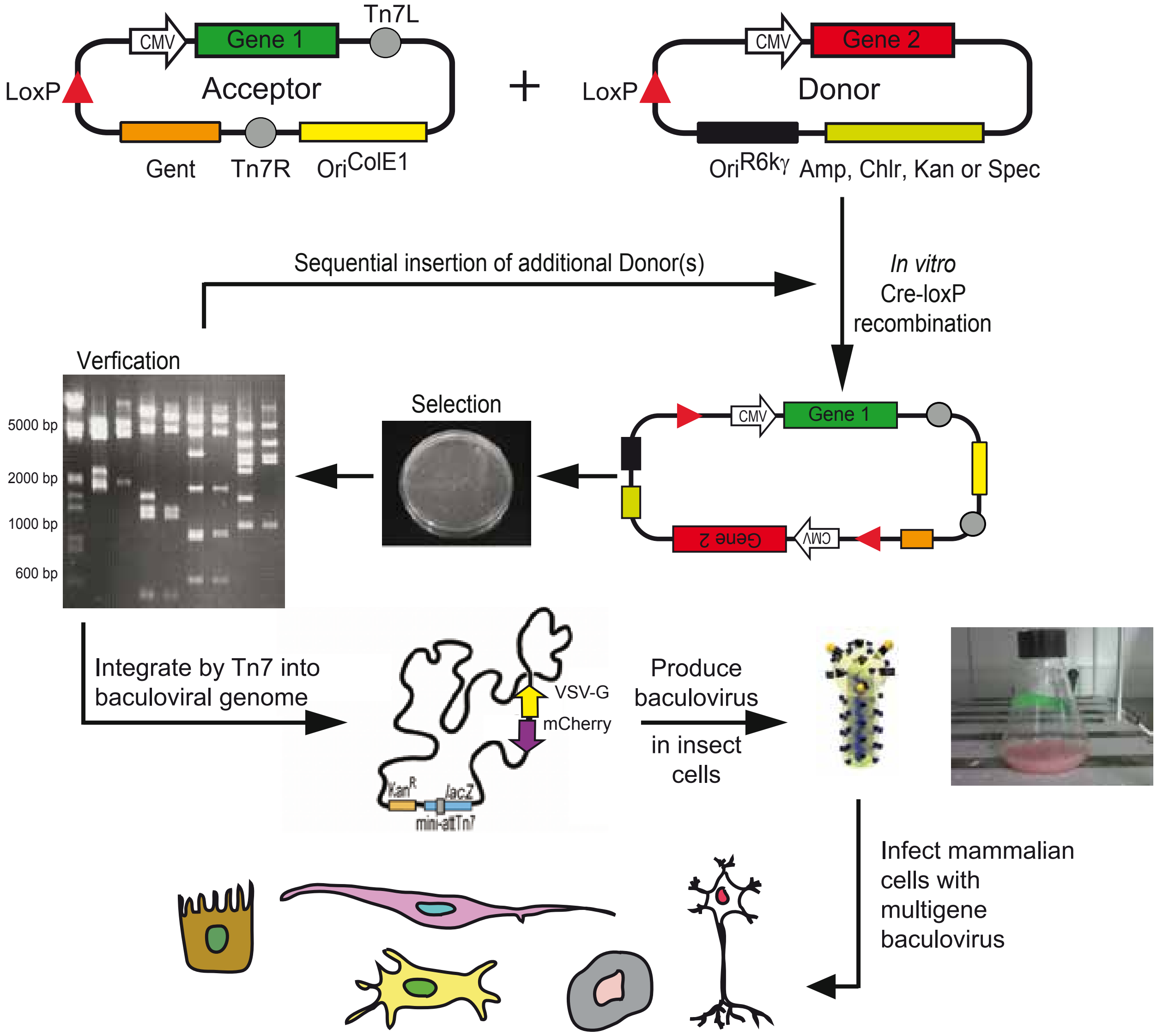

Supplementary Figure 1: Schematic presentation of the MultiPrime workflow.
One Acceptor is fused to one or several Donors (each with a different resistance marker). Acceptor and Donors can each contain one or several genes of interest (one gene shown for clarity). Acceptors contain the Tn7L and Tn7R sequences for transposition mediated by Tn7 transposase. Acceptor-Donor fusions are selected on plates with appropriate antibiotics, and verified by restriction mapping. Cre mediated fusion of further Donors, each with one or several genes of interest, can be performed iteratively. Verified Acceptor-Donor fusions containing all genes and DNA elements (regulatory elements, enhancers, etc) of interest are then integrated into a baculoviral genome (e.g. EMBacY or MultiBacMam) *in vivo* by Tn7 transposition in bacterial cells. Integrands are selected by blue-white screening, and baculovirus produced in insect cell cultures displaying a red or yellow color depending on the used bacmid. MultiBacMam derived baculovirions display a VSV-G protein (yellow spheres) resulting in enhanced infectivity. Recombinant baculovirus is then harvested and can be used to infect a wide range of mammalian cells with high efficiency.
Abbreviations: Gent: Gentamycin; Amp: Ampicillin; Chlr: Chloramphenicol; Kan: Kanamycin; Spec: Spectinomycin; CMV: Cytomegalovirus promoter; Tn7R, Tn7L: Tn7 tranposition elements; loxP: Cre recognition site; ori: origin of replication.

Supplementary Figure 2

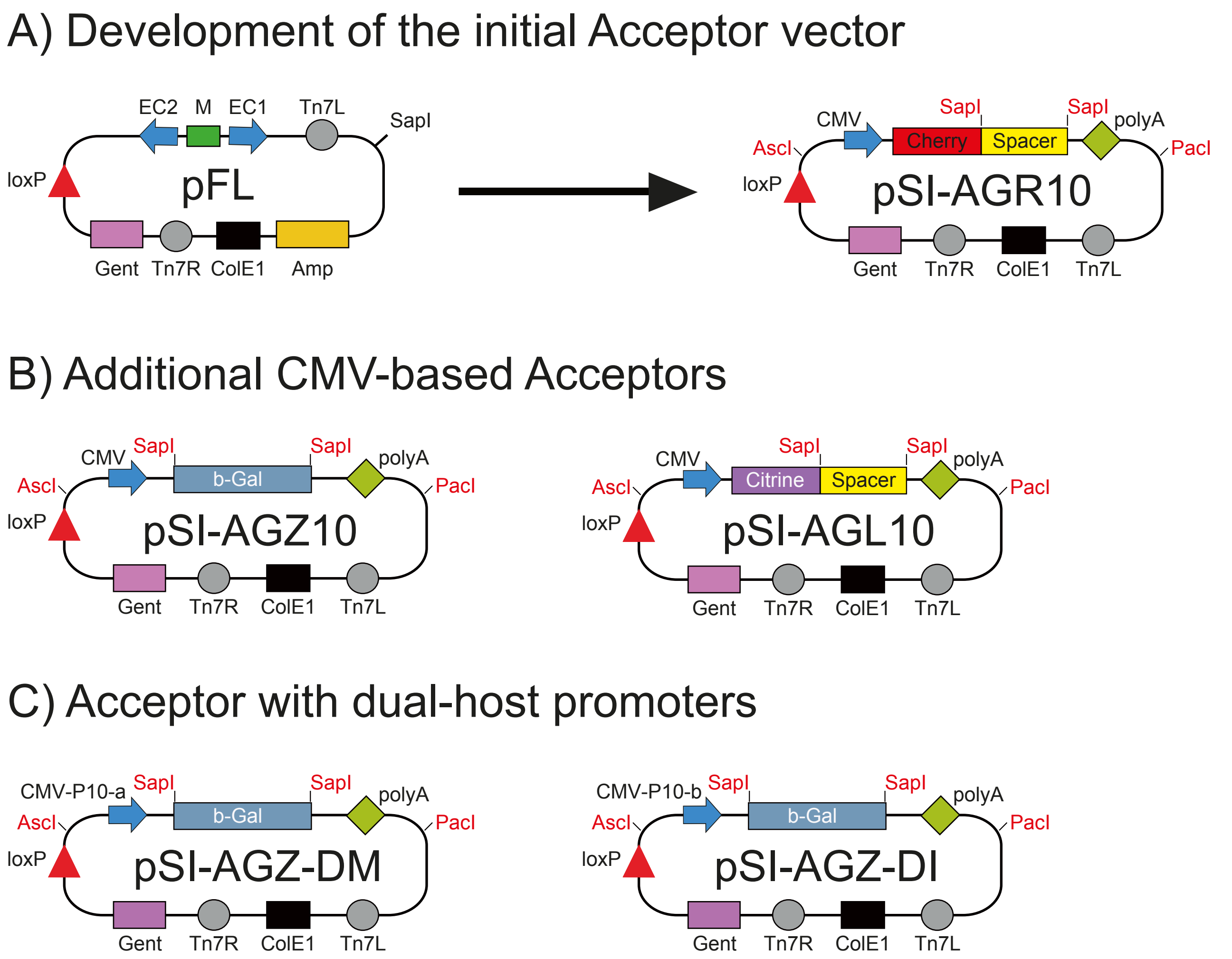

Supplementary Figure 2: MultiPrime compatible Acceptor vectors.
(A) Development of the initial MultiPrime Acceptor vector. This Acceptor plasmid is based on the previously described pFL vector (Fitzgerald et al., 2006). The following changes were made: (i) the ampicillin resistance gene was removed, (ii) an internal SapI site was deleted, and (iii) a mammalian expression cassette was inserted. The mammalian expression cassette derived from the previously described pSI-AKR1 plasmid (Kriz et al., 2010). The cloning sites (red) are compatible within the MultiLabel plasmid family. (B) Additional Acceptors with CMV promoter. (C) Acceptors with dual-host promoters for expression in insect and mammalian cells.
Abbreviations: Gent: Gentamycin; Amp: Ampicillin; M: Multiplication module; EC1,EC2: original pFL expression cassettes; CMV: Cytomegalovirus promoter; polyA: polyadenylation site; lacZ: Marker gene for blue/white screening; Tn7R, Tn7L: Tn7 tranposition elements; loxP: Cre recognition site

Supplementary Figure 3

A) Donors with alternative promoters

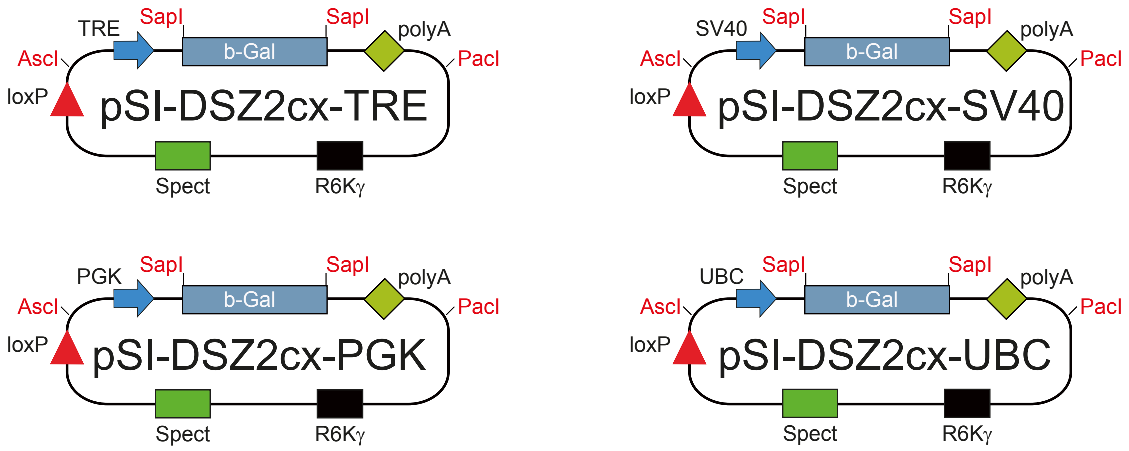

B) Donors with dual-host promoters

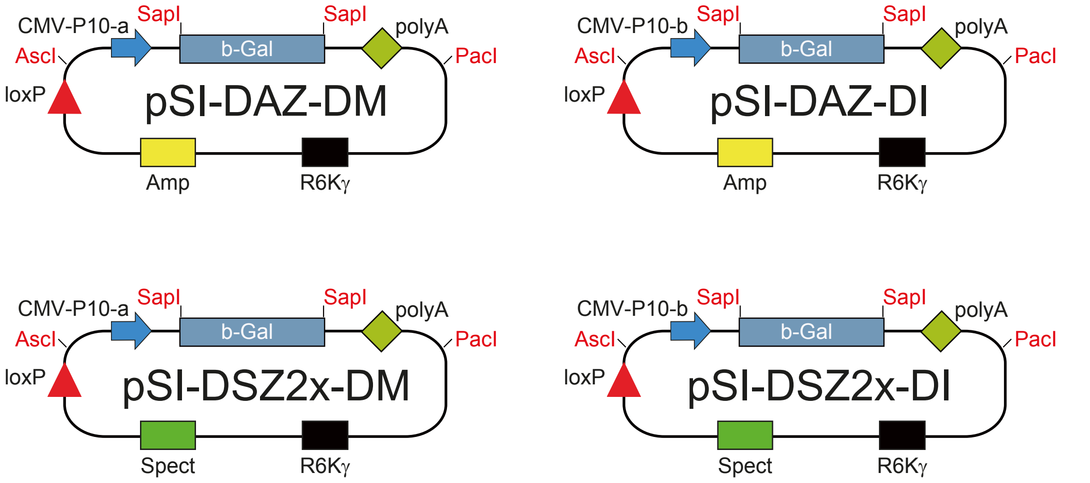

Supplementary Figure 3: MultiPrime compatible Donor vectors.
(A) Donor vectors with alternative promoters for expression in mammalian cells. TRE: tetracycline-inducible promoter; SV40: early promoter from simian virus 40; PGK: phosphoglycerate kinase promoter; UBC: ubiquitin promoter. Please note that all previously described CMV-based promoters are also compatible with MultiPrime (Kriz et al., 2010). (B) Donors with dual-host promoters suitable for insect and mammalian cell expression. Abbreviations: Amp: Ampicillin; Spect: Spectinomycin; b-Gal: spacer with expression of b-galactosidase; R6Kγ: conditional origin of replication; CMV: Cytomegalovirus promoter; polyA: polyadenylation site; loxP: Cre recognition site.

Supplementary Figure 4

Importance of the virus amplification protocol

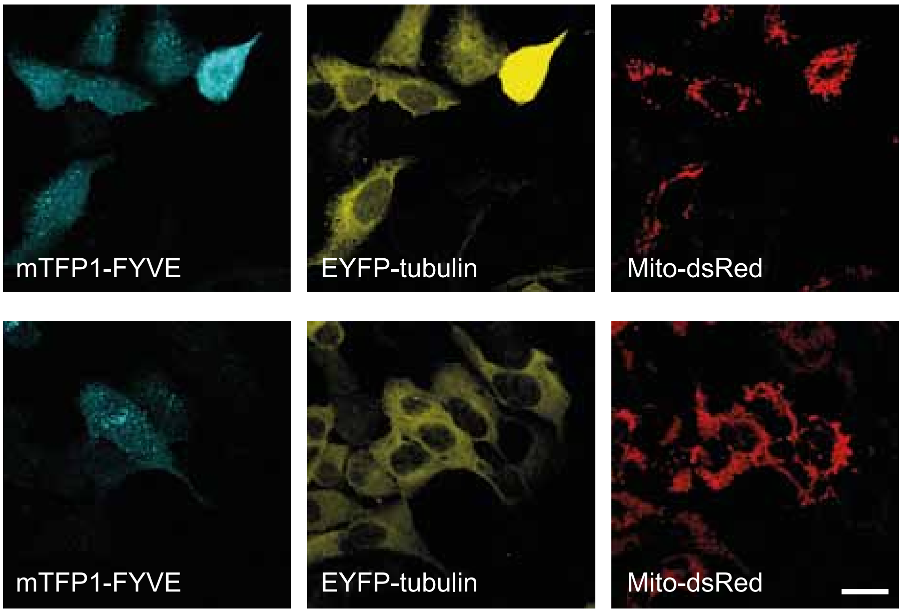

Correct amplification
V0: 50h
V1: 75h
V2: 60h
=> homgenous expression

Inappropriate amplification
V0: 120h
V1: 168h
V2: 144h
=> inhomgenous expression

Supplementary Figure 4: Importance of virus amplification protocol
(Top) Virus amplification according to the protocol in Materials and Methods leads to a homogenous HEK293 cell population. (Bottom) Virus amplification with prolonged incubation times leads to rearrangement of the baculovirus genome and inhomogenous HEK293 cell population after transduction. Scale bar: 20 μm.

Supplementary Figure 5

Additional cell types and constructs

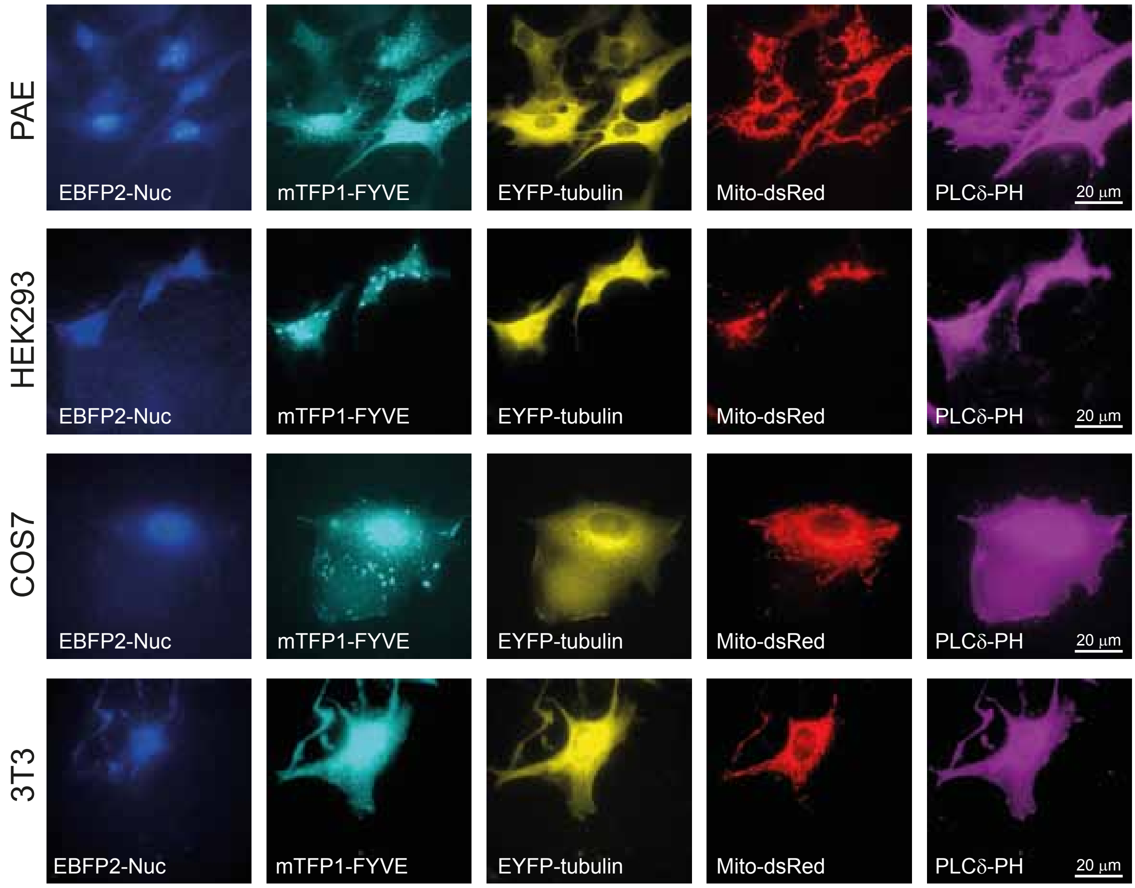

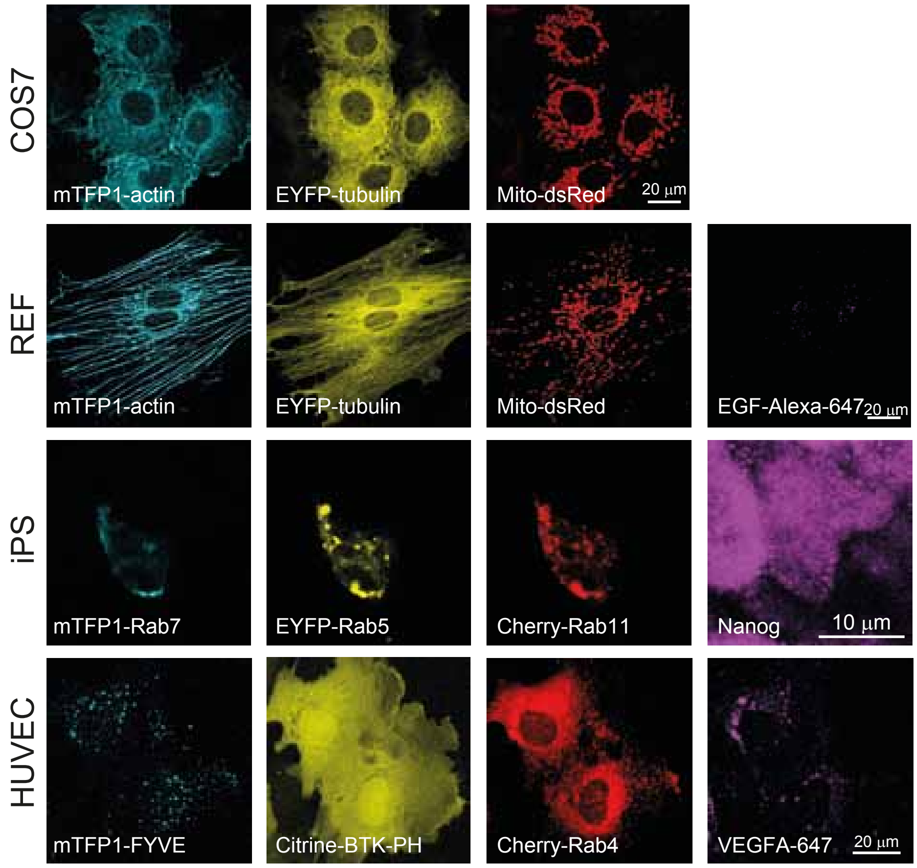

Supplementary Figure 6

Sequences of alternative promoters

Tet responsive promoter (TRE)
GGCGCGCCTAGTTATTAATAGTAATCAATTACGGGGTCGAGCTCGACTTTCACTTTTCTCTATCACTGATAGGGAGTGGTAAACT
CGACTTTCACTTTTCTCTATCACTGATAGGGAGTGGTAAACTCGACTTTCACTTTTCTCTATCACTGATAGGGAGTGGTAAACTC
GACTTTCACTTTTCTCTATCACTGATAGGGAGTGGTAAACTCGACTTTCACTTTTCTCTATCACTGATAGGGAGTGGTAAACTCG
ACTTTCACTTTTCTCTATCACTGATAGGGAGTGGTAAACTCGACTTTCACTTTTCTCTATCACTGATAGGGAGTGGTAAACTCGA
CCTATATAAGCAGAGCTCGTTTAGTGAACCGTCAGATCGCCTGGAGACGCCATCCACGCTGTTTTGACCTCCATAGAAGACACCG
GGACCGATCCAGCCTCCGCGGCCCCGAATTGAAGCTT

Mouse phosphoglycerate kinase (PGK)
GGCGCGCCTAGTTATTAATAGTAATCAATTACGGGGTCTTCTACCGGGTAGGGGAGGCGCTTTTCCCAAGGCAGTCTGGAGCATG
CGCTTTAGCAGCCCCGCTGGGCACTTGGCGCTACACAAGTGGCCTCTGGCCTCGCACACATTCCACATCCACCGGTAGGCGCCAA
CCGGCTCCGTTCTTTGGTGGCCCCTTCGCGCCACCTTCTACTCCTCCCCTAGTCAGGAAGTTCCCCCCCGCCCCGCAGCTCGCGT
CGTGCAGGACGTGACAAATGGAAGTAGCACGTCTCACTAGTCTCGTGCAGATGGACAGCACCGCTGAGCAATGGAAGCGGGTAGG
CCTTTGGGGCAGCGGCCAATAGCAGCTTTGCTCCTTCGCTTTCTGGGCTCAGAGGCTGGGAAGGGGTGGGTCCGGGGGCGGGCTC
AGGGGCGGGCTCAGGGGCGGGGCGGGCGCCCGAAGGTCCTCCGGAGGCCCGGCATTCTGCACGCTTCAAAAGCGCACGTCTGCCG
CGCTGTTCTCCTCTTCCTCATCTCCGGGCCTTTCGACCTAAGCTT

Human Ubiquitin C promoter
GGCGCGCCTAGTTATTAATAGTAATCAATTACGGGGTCGGTGCAGCGGCCTCCGCGCCGGGTTTTGGCGCCTCCCGCGGGCGCCC
CCCTCCTCACGGCGAGCGCTGCCACGTCAGACGAAGGGCGCAGGAGCGTTCCTGATCCTTCCGCCCGGACGCTCAGGACAGCGGC
CCGCTGCTCATAAGACTCGGCCTTAGAACCCCAGTATCAGCAGAAGGACATTTTAGGACGGGACTTGGGTGACTCTAGGGCACTG
GTTTTCTTTCCAGAGAGCGGAACAGGCGAGGAAAAGTAGTCCCTTCTCGGCGATTCTGCGGAGGGATCTCCGTGGGGCGGTGAAC
GCCGATGATTATATAAGGACGCGCCGGGTGTGGCACAGCTAGTTCCGTCGCAGCCGGGATTTGGGTCGCGGTTCTTGTTTGTGGA
TCGCTGTGATCGTCACTTGGTGAGTTGCGGGCTGCTGGGCTGGCCGGGGCTTTCGTGGCCGCCGGGCCGCTCGGTGGGACGGAAG
CGTGTGGAGAGACCGCCAAGGGCTGTAGTCTGGGTCCGCGAGCAAGGTTGCCCTGAACTGGGGGTTGGGGGGAGCGCACAAAATG
GCGGCTGTTCCCGAGTCTTGAATGGAAGACGCTTGTAAGGCGGGCTGTGAGGTCGTTGAAACAAGGTGGGGGGCATGGTGGGCGG
CAAGAACCCAAGGTCTTGAGGCCTTCGCTAATGCGGGAAAGCTCTTATTCGGGTGAGATGGGCTGGGGCACCATCTGGGGACCCT
GACGTGAAGTTTGTCACTGACTGGAGAACTCGGGTTTGTCGTCTGGTTGCGGGGGCGGCAGTTATGCGGTGCCGTTGGGCAGTGC
ACCCGTACCTTTGGGAGCGCGCGCCTCGTCGTGTCGTGACGTCACCCGTTCTGTTGGCTTATAATGCAGGGTGGGGCCACCTGCC
GGTAGGTGTGCGGTAGGCTTTTCTCCGTCGCAGGACGCAGGGTTCGGGCCTAGGGTAGGCTCTCCTGAATCGACAGGCGCCGGAC
CTCTGGTGAGGGGAGGGATAAGTGAGGCGTCAGTTTCTTTGGTCGGTTTTATGTACCTATCTTCTTAAGTAGCTGAAGCTCCGGT
TTTGAACTATGCGCTCGGGGTTGGCGAGTGTGTTTTGTGAAGTTTTTTAGGCACCTTTTGAAATGTAATCATTTGGGTCAATATG
TAATTTTCAGTGTTAGACTAGTAAAGCTT

Simian virus 40 early promoter (SV40)
Derived from pcDNA 3.1 using PCR
GGCGCGCCTAGTTATTAATAGTAATCAATTACGGGGTCCTGTGGAATGTGTGTCAGTTAGGGTGTGGAAAGTCCCCAGGCTCCCC
AGCAGGCAGAAGTATGCAAAGCATGCATCTCAATTAGTCAGCAACCAGGTGTGGAAAGTCCCCAGGCTCCCCAGCAGGCAGAAGT
ATGCAAAGCATGCATCTCAATTAGTCAGCAACCATAGTCCCGCCCCTAACTCCGCCCATCCCGCCCCTAACTCCGCCCAGTTCCG
CCCATTCTCCGCCCCATGGCTGACTAATTTTTTTTATTTATGCAGAGGCCGAGGCCGCCTCTGCCTCTGAGCTATTCCAGAAGTA
GTGAGGAGGCTTTTTTGGAGGCCTAGGCTTTTGCAAAAAGAAGCTT

SV40-forward: GATCGGCGCGCCTAGTTATTAATAGTAATCAATTACGGGGTCCTGTGGAATGTGTGTCAG
SV40-reverse: GATCAAGCTTCTTTTTGCAAAAGCCTAGG

Supplementary Figure 6: Alternative mammalian promoter sequences.
The DNA sequences of promoters used as an alternative to CMV in this study are listed, including the Tet responsive promoter (TRE), the mouse phosphoglycerate kinase promoter (PGK), the human ubiquitin C promoter and the simian virus 40 early promoter (SV40). Insertion of promoters into the MultiPrime plasmids was done usin AscI (red) and HindIII (green).

Supplementary Figure 7

Sequences of dual-host promoters

CMVP10 (based on Philipps B. et al, Biotechnol. Prog., 2005)

Black: CMV sequence
Blue: P10 sequence

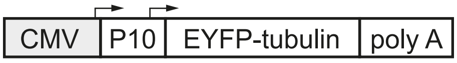

GGCGCGCCTAGTtattaatagtaatcaattacggggtcattagttcatagcccatatatggagttccgcgttacataacttacgg
taaatggcccgcctggctgaccgcccaacgacccccgcccattgacgtcaataatgacgtatgttcccatagtaacgccaatagg
gactttccattgacgtcaatgggtggagtatttacggtaaactgcccacttggcagtacatcaagtgtatcatatgccaagtccg
cccctattgacgtcaatgacggtaaatggcccgcctggcattatgcccagtacatgaccttacgggactttcctacttggcagt
acatctacgtattagtcatcgctattaccatgctgatgcggttttggcagtacaccaatgggcgtggatagcggtttgactcacg
gggatttccaagtctccaccccattgacgtcaatgggagtttgttttggcaccaaaatcaacgggactttccaaaatgtcgtaat
aaccccgccccgttgacgcaaatgggcggtaggcgtgtacggtgggaggtctatataagcagacgtcgtttagtgaaccgtcaga
tcaccatccagtatacggacctttaattcaacccaacacaatatattatagttaaataagaattattatcaaatcatttgtatat
taattaaaatactatactgtaaattacattttatttacaatcactcgacgaagacttgatcacccgggatctcgaAAGCTT

CMVintP10 (including an intron with the P10 promoter)

Black: CMV sequence
Blue: P10 sequence
yellow: intron sequence

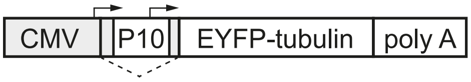

GGCGCGCCTAGTTATTAATAGTAATCAATTACGGGGTCATTAGTTCATAGCCCATATATGGAGTTCCGCGTTACATAACTTACGG
TAAATGGCCCGCCTGGCTGACCGCCCAACGACCCCCGCCCATTGACGTCAATAATGACGTATGTTCCCATAGTAACGCCAATAGG
GACTTTCCATTGACGTCAATGGGTGGAGTATTTACGGTAAACTGCCCACTTGGCAGTACATCAAGTGTATCATATGCCAAGTACG
CCCCCTATTGACGTCAATGACGGTAAATGGCCCGCCTGGCATTATGCCCAGTACATGACCTTATGGGACTTTCCTACTTGGCAGT
ACATCTAGTATTAGTCATCGCTATTACCATGGTGATGCGGTTTTGGCAGTACATCAATGGGCGTGGATAGCGGTTTGACTCACGG
GGATTTCCAAGTCTCCACCCCATTGACGTCAATGGGAGTTTGTTTTGGCACCAAAATCAACGGGACTTTCCAAAATGTCGTAACA
ACTCCGCCCCATTGACGCAAATGGGCGGTAGGCGTGTACGGTGGGAGGTCTATATAAGCAGAGCTCGTTTAGTGAACCGTCAGAT
CGCCTGGAGACGCCATCCACGCTGTTTTGACCTCCATAGAAGACACCGGGACCGATCCAGCCTCCGCGGCCGGGAACGGTGCATT
GGAACGCGGATTCCCCGTGCCAAGAGTGACGTAAGTACCGCCTATAGACTCTATAGGCACACCCCTTTGGCTCTTggacctttaa
ttcaacccaacacaatatattatagttaaaTAAGaattattatcaaatcatttgtatattaattaaaatactatactgtaaatta
cattttatttacaatcAGACAGACTGTTCCTTTCCTGGGTCTTTTCTGCAGGCACCGTCGTCGACTTAACAGATCTGTTTAAGCT
T

Supplementary Figure 7: Dual-host promoter sequences for expression in insect and mammalian cells.
The DNA sequences of dual-host promoters CMVP10 and CMVintP10 are shown, which support heterologous expression of target proteins of choice both in insect cells and in mammalian cells. Promoter structures are depicted in a schematic representation. Promoter DNAs were inserted into MultiPrime Acceptor and Donors by using restriction sites AscI (red letters) and HindIII (green letters). CMV stands for cytomegalovirus promoter, P10 for baculoviral very late promoter p10, polyA for the SV40 polyadenylation site. CMVintP10 contains in addition an intron sequence.

Supplementary Figure 8

Human transcription factor complex expression

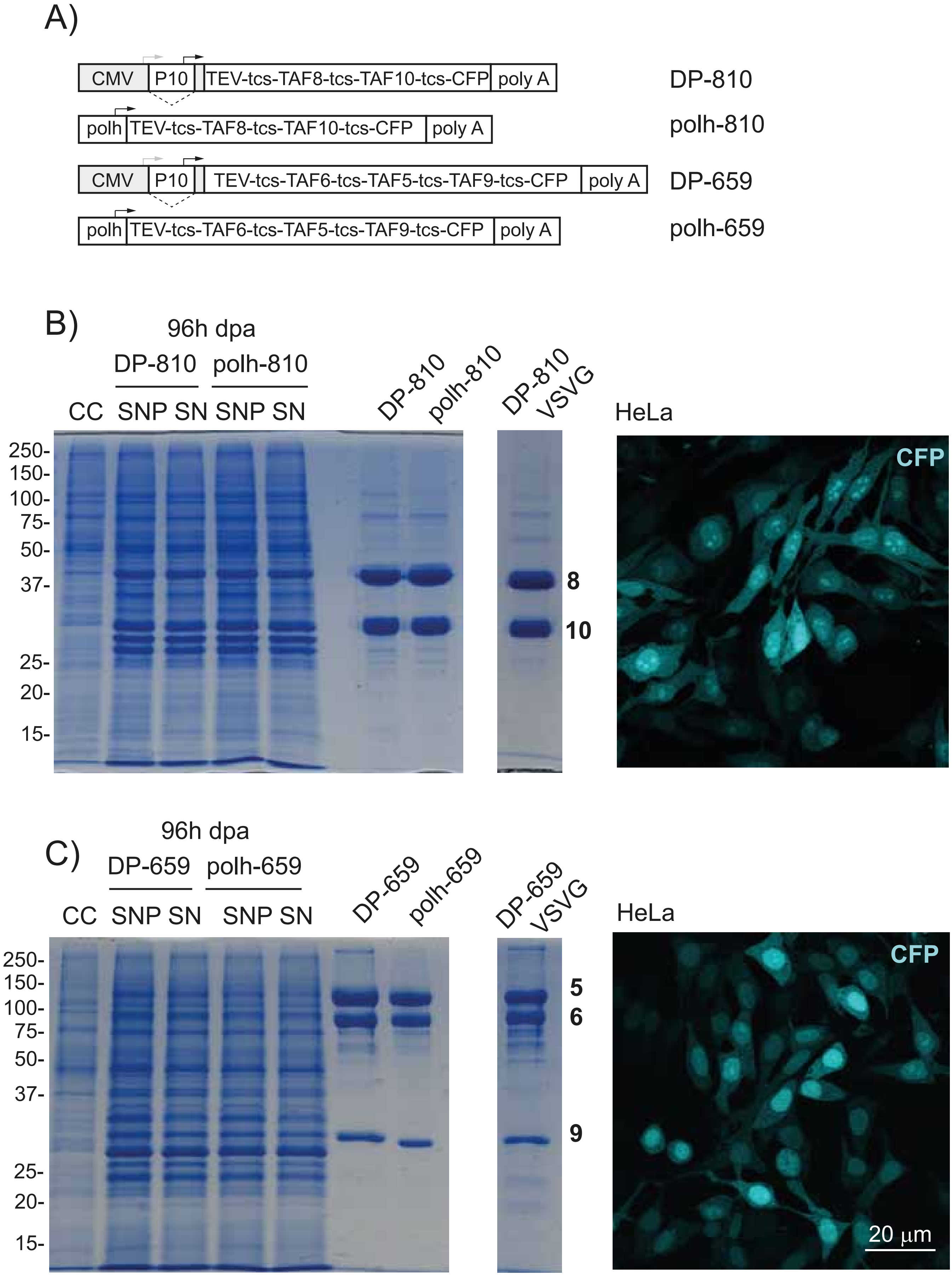

Supplementary Figure 8: Validation of bifunctional dual promoter expression cassettes. Human General Transcription (GTF) factor TFIID subcomplex expression from expression cassettes driven by the bifunctional dual promoter CMVintP10-b was compared to expression from baculoviral very late promoter polyhedrin (polh) driven cassettes which are only active in insect cells. (**A**) Expression cassettes tested are shown in a schematic representation. CMV stands for cytomegalovirus late promoter, P10 for baculoviral very late promoter p10. TEV stands for tobacco etch virus NIa protease, tcs for TEV cleavage site, TAF stands for TBP associated factor, CFP for cyan fluorescent protein and poly A for the SV40 polyadenylation site. Small arrows indicate transcription start sites. An intron sequence enhancing transcription following the p10 promoter is represented as a box colored in gray. TFIID subcomplexes TAF8-TAF10 and TAF5-TAF6-TAF9 were expressed as self-processing polyproteins tailored into the individual subunits by TEV. (**B**) SDS-PAGE analysis of the expression of TAF8-TAF10 complex in baculovirus-infected insect cell cultures is shown (left). CC stands for uninfected cell control, SNP for whole cell extract, SN for cleared lysate. DP-810 denotes expression from the bifunctional dual promoter, polh-810 denotes expression from the baculoviral late promoter. EMBacY baculovirus was used. Virtually identical amounts of purified TAF8-TAF10 complex were obtained in the expression experiments. DP-810 VSVG denotes purified TAF8-TAF10 complex expressed from the bifunctional promoter, using MultiBacMam virus which displays VSV-G protein on its surface to enhance mammalian cell infectivity (middle). This virus was used to efficiently infect HeLa cells as evidenced by CFP fluorescence (right). (**C**) SDS-PAGE analysis of the expression of TAF5-TAF6-TAF9 complex in baculovirus-infected insect cell cultures is shown (left). CC stands for uninfected cell control, SNP for whole cell extract, SN for cleared lysate. DP-659 denotes expression from the bifunctional dual promoter, polh-659 denotes expression from the baculoviral late promoter. EMBacY baculovirus was used. Virtually identical amounts of purified TAF5-TAF6-TAF9 complex were obtained in the expression experiments. DP-659 VSVG denotes purified TAF5-TAF6-TAF9 complex expressed from the bifunctional promoter, using MultiBacMam virus which displays VSV-G protein on its surface to enhance mammalian cell infectivity (middle). This virus was used to infect HeLa cells with high efficiency as evidenced by CFP fluorescence (right).

Supplementary Figure 9
Modification of the HMGA1 locus with CRISPR

modified from Ratz et al., Scientific Reports, 2015
(EGFP instead of rsEGFP2 was used)

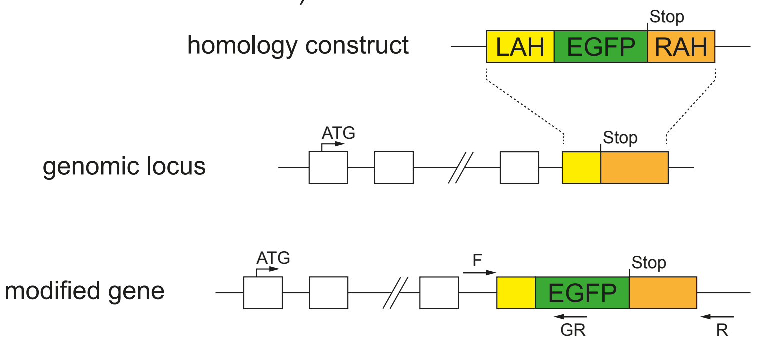

HMGA1_out_F: GTCCCTCCCTCTCCTGCTCCTAGAATACTCAG
HMGA1_out_F3: TTGTCCTTCCTATGAGCCTCTGCAG
HMGA1_out_R: CAATGACGGATGTCGAAGAATGGAACATTGAAC
EGFP_R: CACGCTGCCGTCCTCGATGTTG
EGFP-N-Rev-long: GTCAGCTTGCCGTAGGTGGCATC

Plasmids for modification of the human HMGA1 locus
Plasmids were obtained from Stefan Jakobs (Ratz et al., 2015). The homology arm was cloned into the AscI/ PacI sites of AG10. rsEGFP2 was replaced by EGFP (left). U6-driven HMGA1-gRNA2 and Cas9 expression cassette from px330 were transfered to AscI/ PacI sites of plasmid DA2 (middle). U6-driven HMGA1-gRNA1 from px330 was transfered to AscI/ PacI sites of plasmid DS2cx (right).

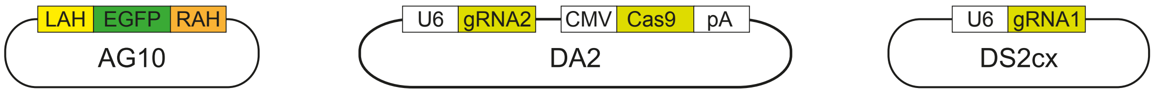

Baculovirus constructs for modification of the human HMGA1 locus

Homology construct („Homology“ in figures)

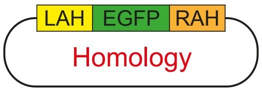

Homology construct / Cas9 - gRNA2 („gRNA2“ in figures)

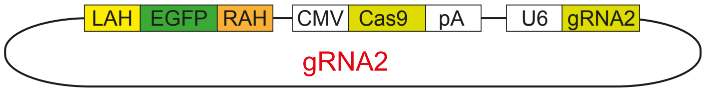

Homology construct / Cas9 - gRNA2 / gRNA1 („gRNA 1+2“ in figures)

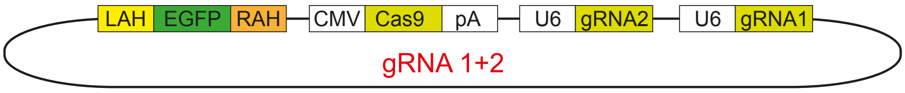

MultiPrime-based genome engineering of HEK293 cells. (left) HEK293 cells were infected with the indicated viruses to modify the HMGA1 locus. Cells were fixed after five days and the nuclei were counterstained with NucRed Dead 647 (Molecular Probes, red). Cells with a modified genome have a green nuclei. Scale bar: 20 µm. (right) PCR analysis of the infected cultures. The fragment with 1139bp for the mutant allel was only obtained from cultures that express the Homology construct together with gRNAs and Cas9.

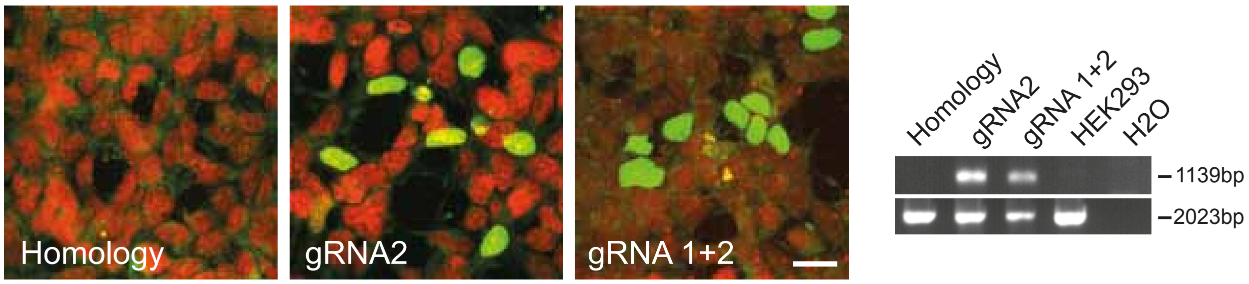

Supplementary Figure 10a

i) Original western blots of HEK293 cells (modulation of expression levels)

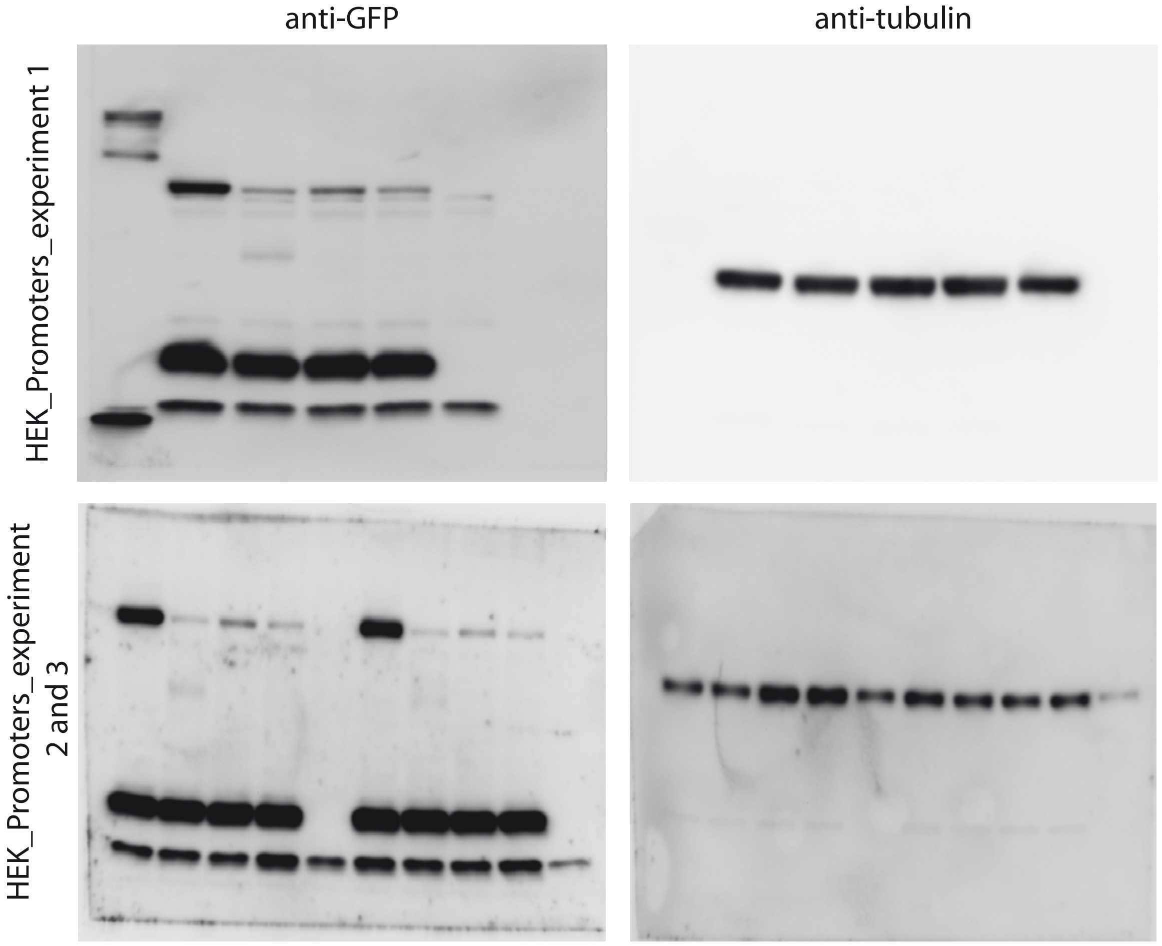

ii) Original western blots of HEK293 cells (dual-host promoters)

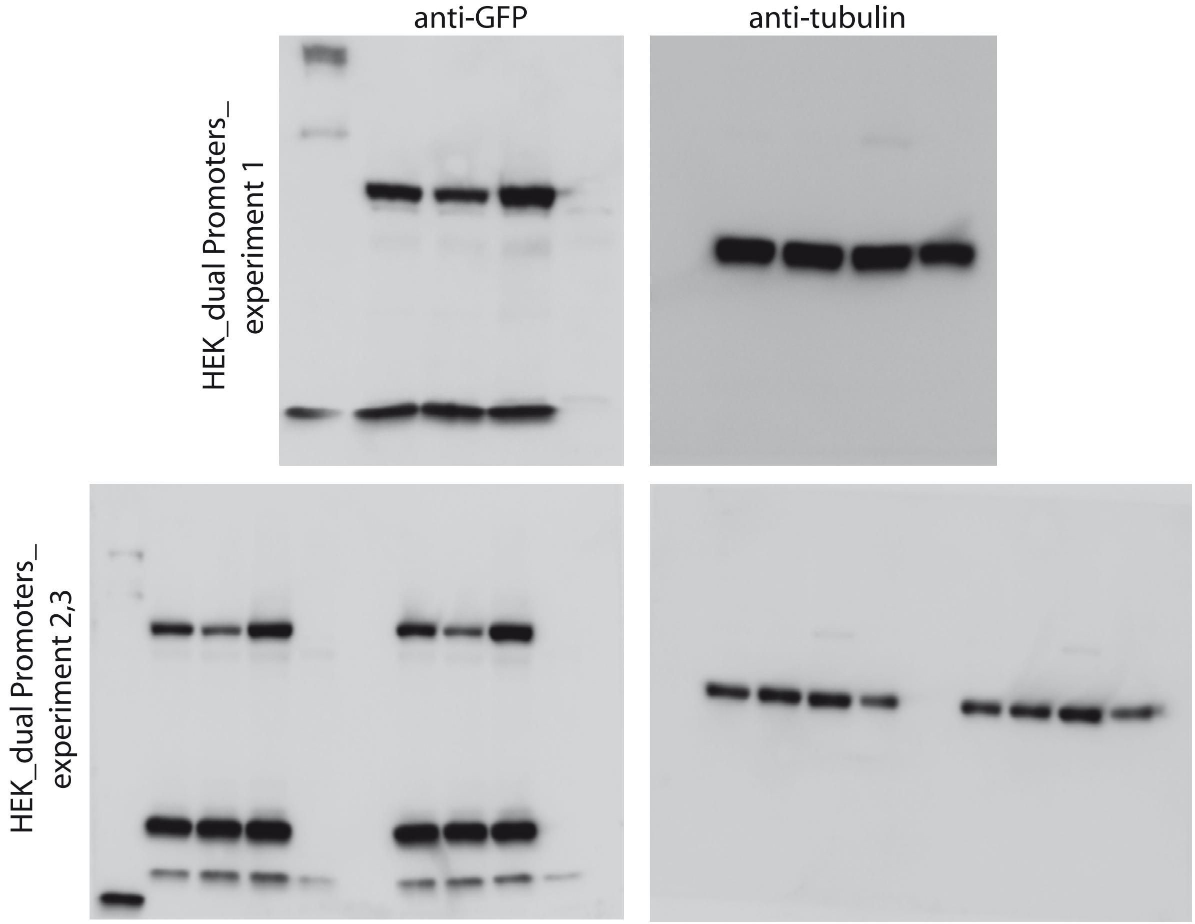

Supplementary Figure 10b

Original western blots of REF cells (modulation of expression levels and dual-host promoters)

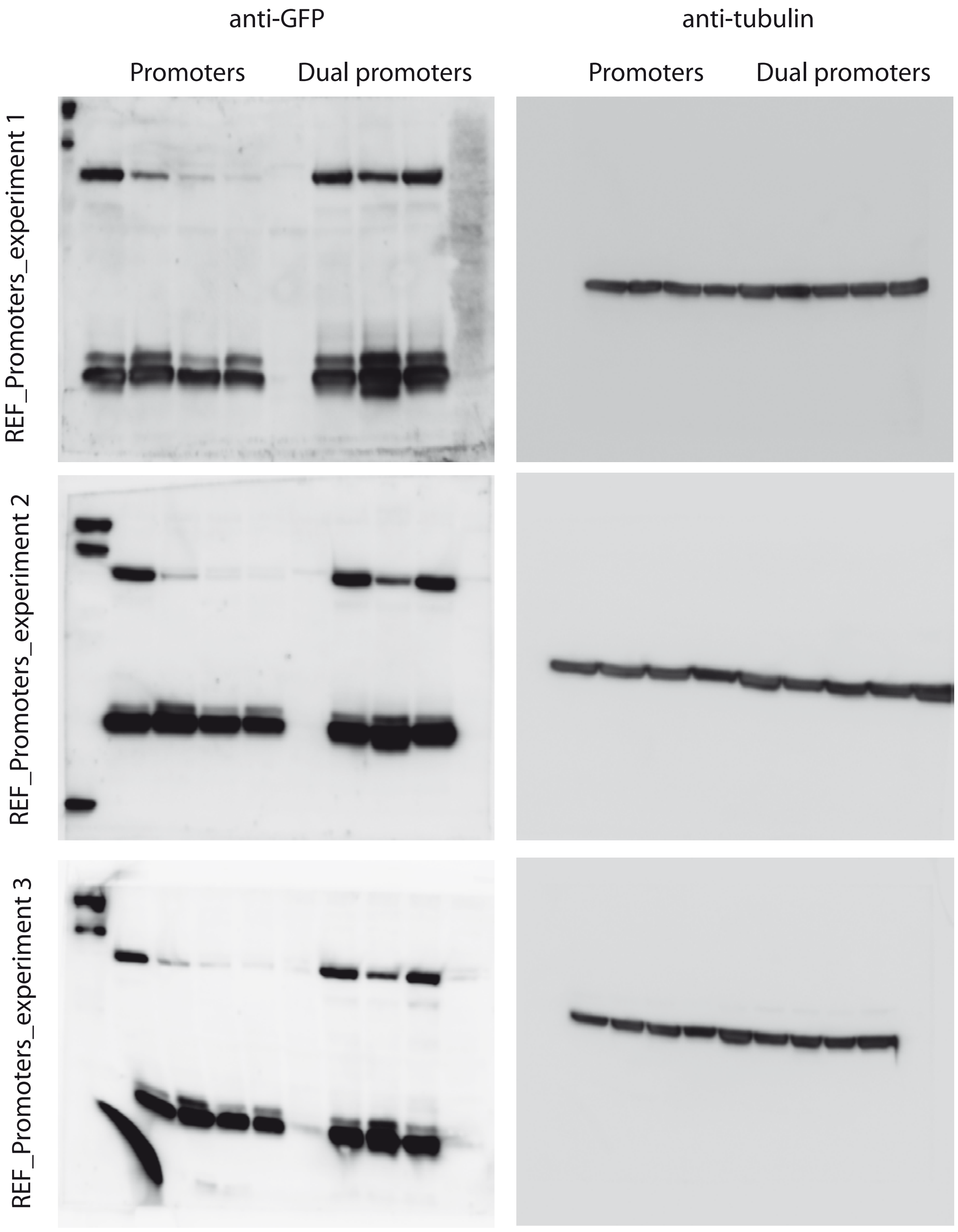

Supplementary Figure 10c

Original western blots of PAE cells (modulation of expression levels and dual-host promoters)

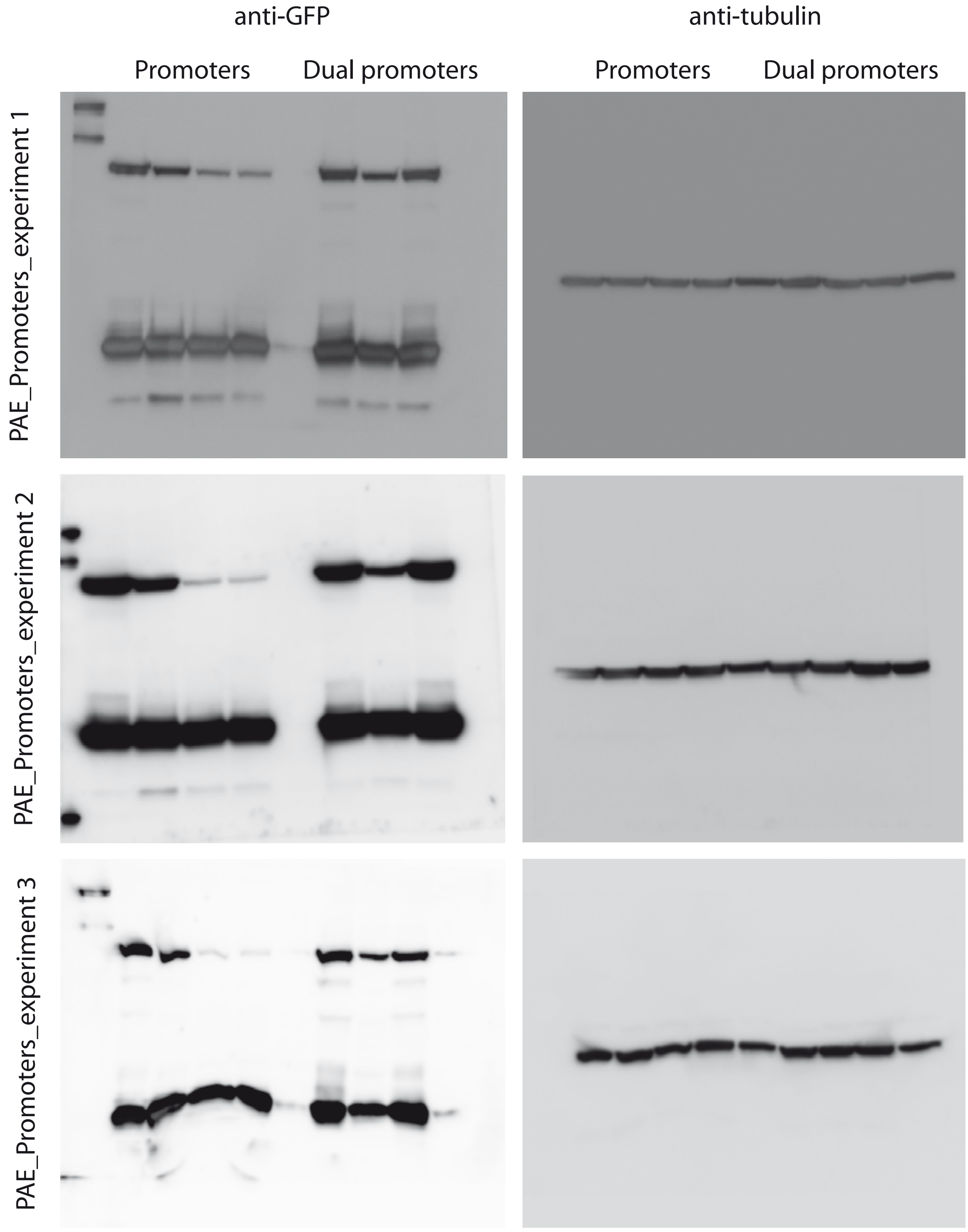

Supplementary Figure 10d

i) Original western blots of Hela tTA cells (modulation of expression levels)

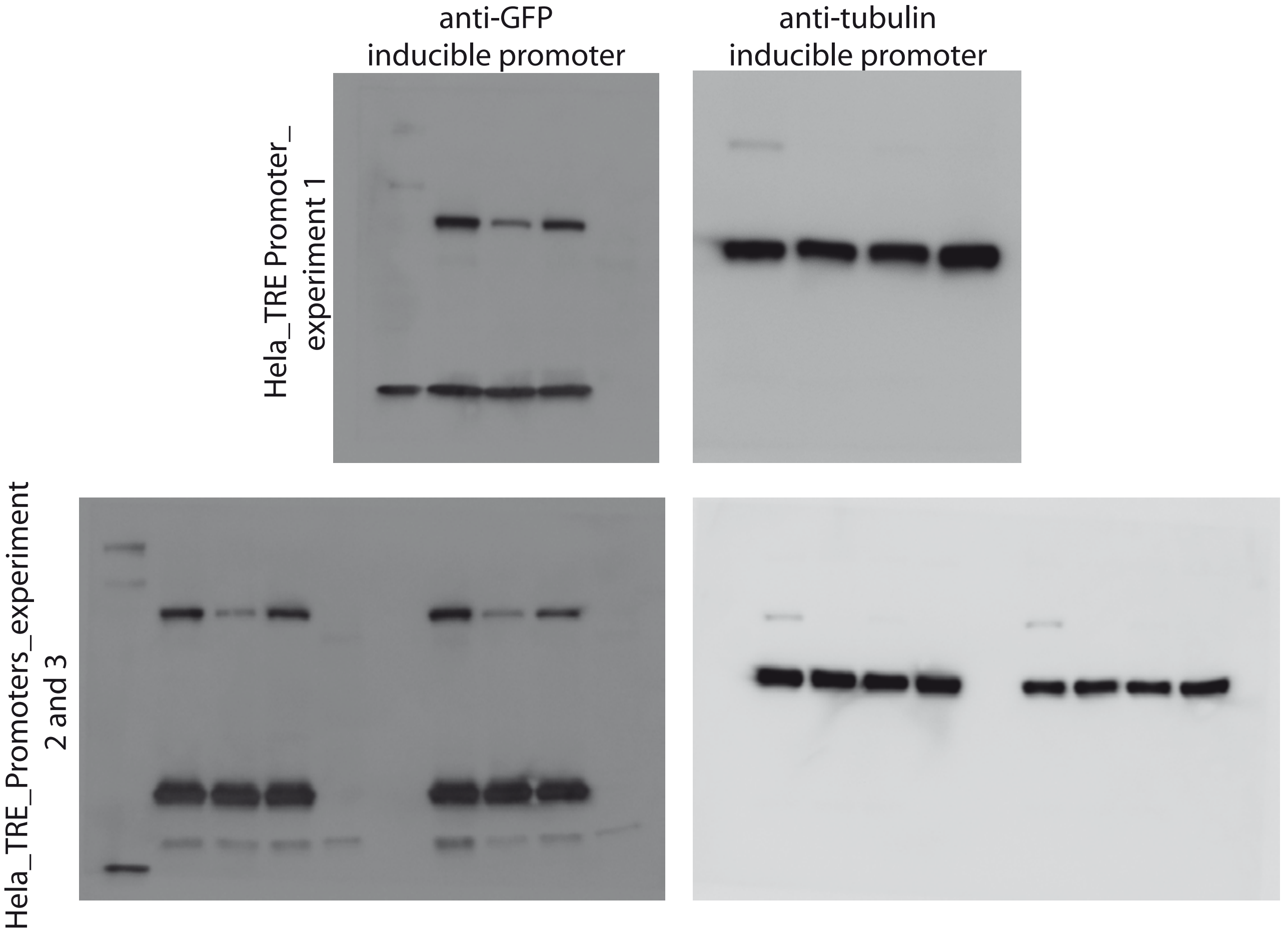

ii) Original western blots of Sf21(insect) cells (dual-host promoters)

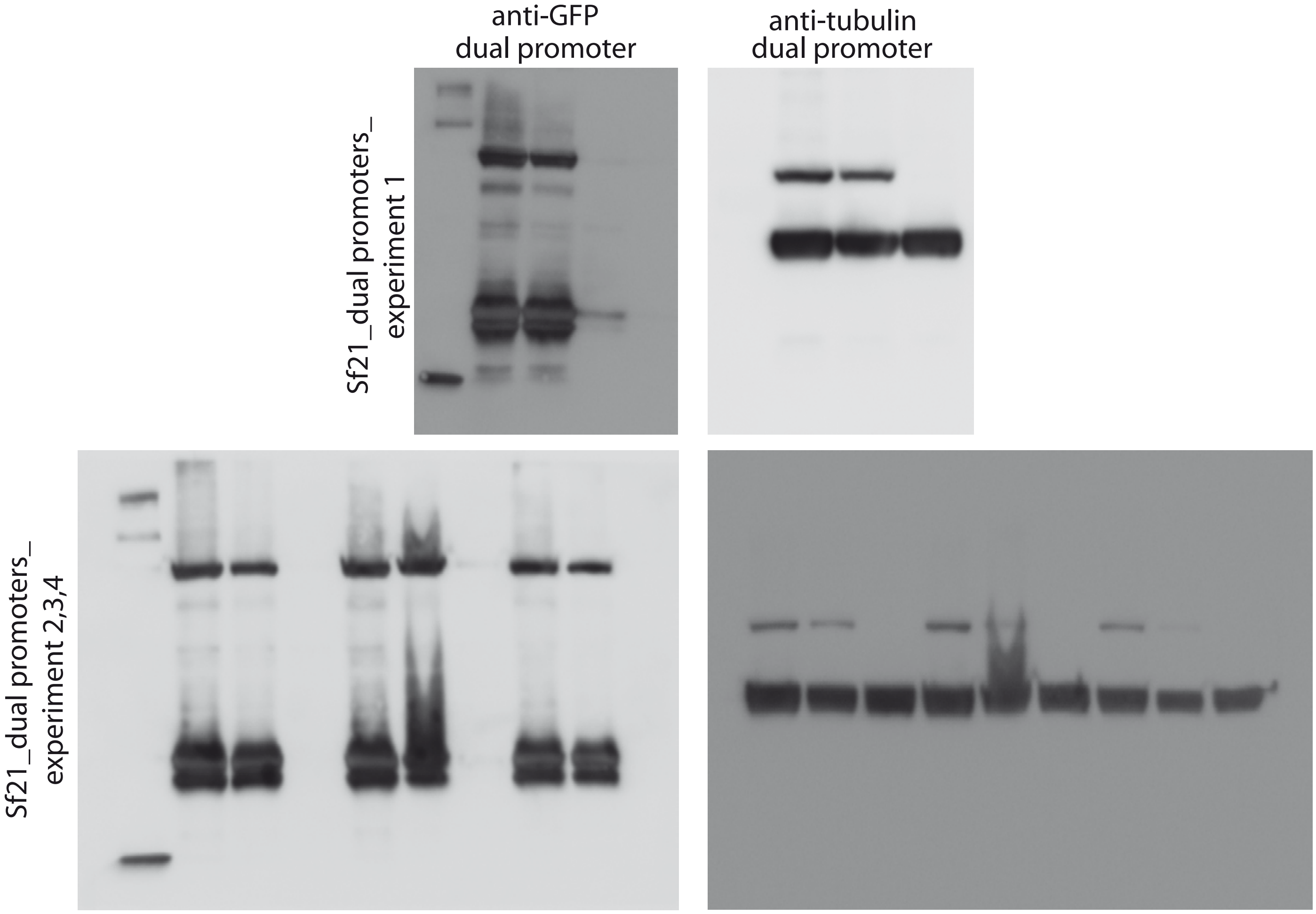

Supplementary Figure 10e

Original western blots related to conversion of single chains to full length human antibodies

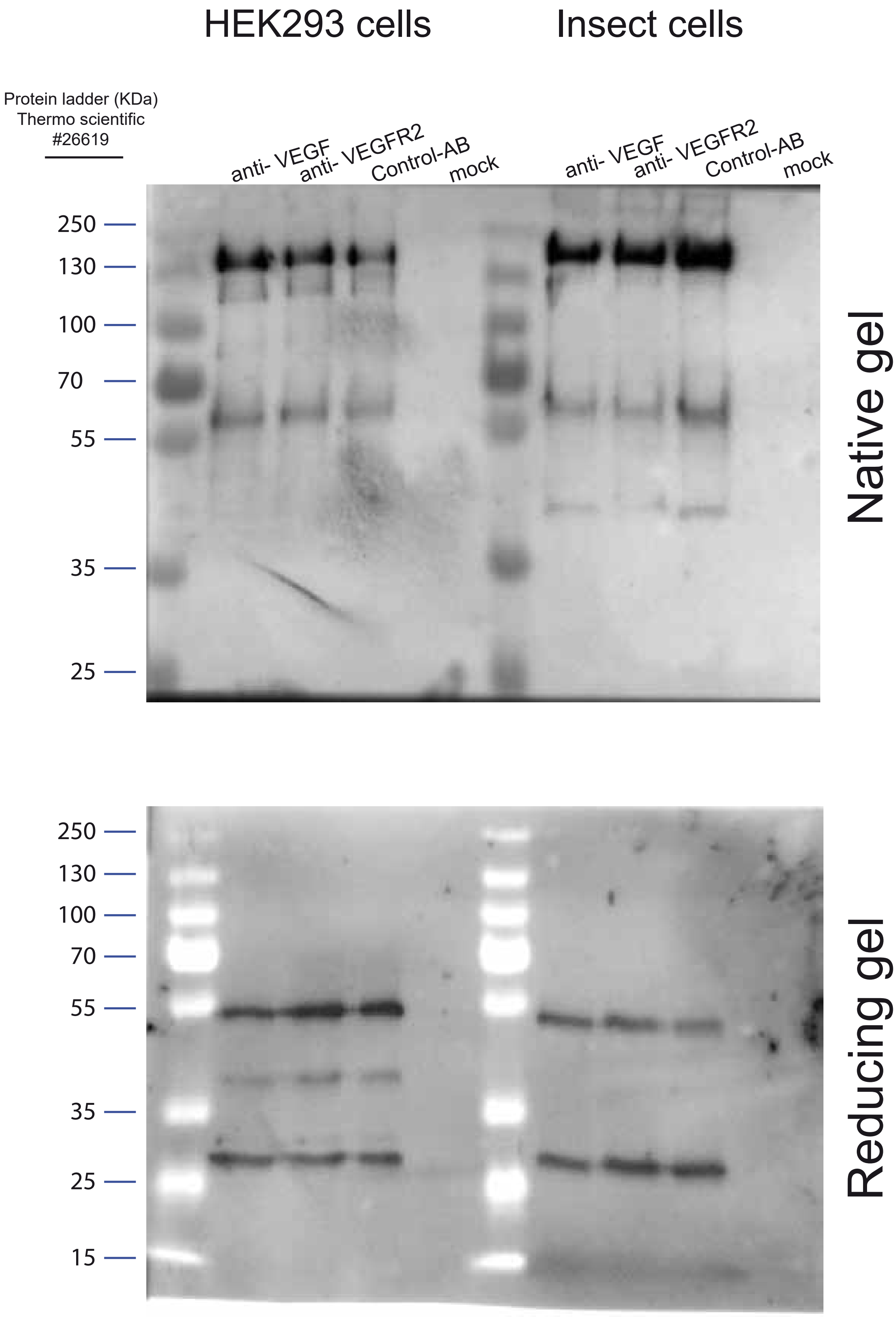

Supplement: Supplementary Information — Supplementary Figures 1-10 [file ncomms11529-s1.pdf]
