# Supplementary material for: Highly efficient baculovirus-mediated multigene delivery in primary cells
Source: Nat Commun. 2016 May 4;7:11529. doi: 10.1038/ncomms11529 (PMC4857464; doi:10.1038/ncomms11529)
Supplement: Peer review file [file ncomms11529-s4.pdf]

Transferred manuscripts:

Editorial Note: this manuscript has been previously reviewed at another journal that is not operating a transparent peer review scheme. This document only contains reviewer comments and rebuttal letters for versions considered at Nature Communications.
